# Characterising the treatment of thromboembolic events after COVID-19 vaccination in 4 European countries and the US: An international network cohort study

**DOI:** 10.3389/fphar.2023.1118203

**Published:** 2023-03-24

**Authors:** Aniek F. Markus, Victoria Y. Strauss, Edward Burn, Xintong Li, Antonella Delmestri, Christian Reich, Can Yin, Miguel A. Mayer, Juan-Manuel Ramírez-Anguita, Edelmira Marti, Katia M. C. Verhamme, Peter R. Rijnbeek, Daniel Prieto-Alhambra, Annika M. Jödicke

**Affiliations:** ^1^ Department of Medical Informatics, Erasmus University Medical Center, Rotterdam, Netherlands; ^2^ Centre for Statistics in Medicine (CSM), Nuffield Department of Orthopaedics, Rheumatology and Musculoskeletal Sciences (NDROMS), University of Oxford, Oxford, United Kingdom; ^3^ Fundació Institut Universitari per a la recerca a l’Atenció Primària de Salut Jordi Gol i Gurina (IDIAPJGol), Barcelona, Spain; ^4^ Real World Solutions, IQVIA, Durham, NC, United States; ^5^ Hospital del Mar Medical Research Institute (IMIM), Universitat Pompeu Fabra, Parc de Salut Mar, Barcelona, Spain; ^6^ Hematology Department. Hospital Clínico Universitario de Valencia, Valencia, Spain; ^7^ Department of Bioanalysis, Ghent University, Ghent, Belgium

**Keywords:** treatment pathways, drug utilization, thromboembolic events, vaccination, anticoagulation, epidemiology, COVID-19

## Abstract

**Background:** Thrombosis with thrombocytopenia syndrome (TTS) has been identified as a rare adverse event following some COVID-19 vaccines. Various guidelines have been issued on the treatment of TTS. We aimed to characterize the treatment of TTS and other thromboembolic events (venous thromboembolism (VTE), and arterial thromboembolism (ATE) after COVID-19 vaccination and compared to historical (pre-vaccination) data in Europe and the US.

**Methods:** We conducted an international network cohort study using 8 primary care, outpatient, and inpatient databases from France, Germany, Netherlands, Spain, The United Kingdom, and The United States. We investigated treatment pathways after the diagnosis of TTS, VTE, or ATE for a pre-vaccination (background) cohort (01/2017—11/2020), and a vaccinated cohort of people followed for 28 days after a dose of any COVID-19 vaccine recorded from 12/2020 onwards).

**Results:** Great variability was observed in the proportion of people treated (with any recommended therapy) across databases, both before and after vaccination. Most patients with TTS received heparins, platelet aggregation inhibitors, or direct Xa inhibitors. The majority of VTE patients (before and after vaccination) were first treated with heparins in inpatient settings and direct Xa inhibitors in outpatient settings. In ATE patients, treatments were also similar before and after vaccinations, with platelet aggregation inhibitors prescribed most frequently. Inpatient and claims data also showed substantial heparin use.

**Conclusion:** TTS, VTE, and ATE after COVID-19 vaccination were treated similarly to background events. Heparin use post-vaccine TTS suggests most events were not identified as vaccine-induced thrombosis with thrombocytopenia by the treating clinicians.

## Introduction

COVID-19 vaccines have shown great effectiveness in preventing severe COVID-19 disease, a disease which lead to hospitalizations and related deaths (Polack et al., 2020; Baden et al., 2021; Voysey et al., 2021). However, after millions of doses were administered in large-scale immunization campaigns, reports of a rare but potentially life-threatening adverse event combining thrombosis with thrombocytopenia, also known as vaccine-associated immune thrombosis and thrombocytopenia (VITT)—Following vaccination with viral vector-based vaccines emerged (Cines and Bussel, 2021; Greinacher et al., 2021; Schultz et al., 2021). Due to difficulties in the diagnosis and identification of VITT, most related research has used thrombosis with thrombocytopenia syndrome (TTS) as a proxy for the study of vaccine safety (Hippisley-Cox et al., 2021), although careful evaluation of TTS suggests it does not match the profile of VITT cases (Shoaibi et al., 2022).

TTS following vaccination is rare, with reported incidences of 14.9 per million and 1.8 cases per million, after a first or second vaccine dose (Pavord et al., 2021). As for its pathogenesis, an immune response leading to the development of pathological anti-platelet factor 4 (anti-PF4) antibodies after vaccination was suggested (Cines and Bussel, 2021; Pavord et al., 2021; Scully et al., 2021), which then leads to activation of platelets and the coagulation system. TTS presents as thrombosis in the cerebral veins in half of the cases, but other arterial or venous vessels can also be affected. Because of concerns of secondary hemorrhage, TTS are challenging to manage in clinical practice (Pavord et al., 2021).

Various guidelines have therefore been issued on the management of TTS following COVID-19 vaccination (Gresele et al., 2021; Oldenburg et al., 2021; Pavord et al., 2021; Rizk et al., 2021; World Health Organization, 2021). The World Health Organisation (WHO) recommends using intravenous immunoglobulins (IVIG) and/or non-heparin-based anticoagulants in people with TTS after vaccination, while platelet transfusions should be avoided, except for emergency situations that require surgery (World Health Organization, 2021). As pathogenesis of VITT resembles that of heparin-induced thrombocytopenia (HIT), treatment should be similar to it and heparins should be avoided in individuals with high clinical suspicion or positive anti-PF4 antibodies) (Gresele et al., 2021; Oldenburg et al., 2021). However, those guidelines were only published throughout the roll-out of the vaccination campaigns, and hence were not available to clinicians in the early days of vaccination.

In response to the TTS safety signal, large-scale observational studies not only assessed the risk of TTS but also assessed the association of COVID-19 vaccines and thromboembolic events (TE) without concurrent thrombocytopenia. Compared to the expected rates in the background population, significantly higher incidence rates were reported for pulmonary embolism (PE) following vaccination with ChAdOx1 or BNT162b (Burn et al., 2022a; Burn et al., 2022b). Another study based on Danish and Norwegian data reports increased rates of venous thromboembolism (VTE), PE and cerebral venous sinus thrombosis (CVST) following ChAdOx (Pottegard et al., 2021; Burn et al., 2022a). However, risk for thromboembolic events was substantially higher following SARS-CoV-2 infection compared to vaccination (Burn et al., 2022a; Burn et al., 2022b), which highlights the burden of thromboses in the context of the COVID-19 pandemic.

As part of a project requested and funded by the European Medicines Agency (EMA) to investigate the association between thrombosis with thrombocytopenia syndrome (TTS) or thromboembolic events and COVID-19 vaccines, we aimed to characterize the treatment of TTS, VTE, or arterial thromboembolism (ATE) after COVID-19 vaccination compared with a pre-vaccination cohort.

## Materials and methods

### Data sources

We conducted an international network cohort study using routinely collected health data mapped to the Observational Medical Outcomes Partnership Common Data Model (OMOP CDM) (Overhage et al., 2012). Data was obtained from primary care, outpatient, and inpatient databases from France, Germany, Netherlands, Spain, The United Kingdom, and The United States. A summary of key features of the data sources is reported in Table 1.

**TABLE 1:** List of databases included in study.

Database abbreviation	Database name	Country	Key data available				
			COVID vaccines	Hospital treatments	Hospital outcomes	Outpatient treatments	Platelet counts
**CPRD**	Clinical Practice Research Datalink AURUM	United Kingdom	Complete	No	Incomplete	Yes	Yes
**SIDIAP**	Information System for Research in Primary Care CMBD	Spain	Complete	No	Yes	Yes	Yes
**IPCI**	Integrated Primary Care Information	Netherlands	Incomplete	No	Incomplete	Yes	Yes
**LPD France**	Longitudinal Patients Database France	France	Incomplete	No	Incomplete	Yes	Yes
**DA Germany**	IQVIA Data Analyzer Germany	Germany	Incomplete	No	Incomplete	Yes	Yes
**Open Claims**	IQVIA Open Claims	United States	Incomplete	Incomplete	Incomplete	Yes	Yes
**Hospital CDM**	Hospital Charge Data Master CDM	Unites States	Incomplete	Yes	Yes	Incomplete	Incomplete
**IMASIS**	Parc de Salut Mar Information System	Spain	Incomplete	Yes	Yes	No	Yes

Outpatient and inpatient databases provide complementary information on the management of TTS, VTE and ATE. Treatment typically starts in-hospital following emergency department admission, but usually continues in outpatient setting for secondary prevention. Therefore, our study was based on different databases, reflecting different settings and healthcare systems: Unadjudicated health claims (Open Claims) and hospital electronic medical records (Hospital CDM and IMASIS) from the US/Spain contributed inpatient data. Since general practitioners act as gatekeepers to the healthcare system in Netherlands, Spain and the United Kingdom, primary care databases (IPCI, SIDIAP, and CPRD, respectively) are best positioned to identify COVID-19 vaccines and to provide information on health outcomes in these countries. For those European countries where general practitioners do not act as gatekeepers to the system, such as France and Germany, outpatient records (LPD France and DA Germany) including general practice and ambulatory specialist data were used.

Vaccine exposure was completely recorded in CPRD and SIDIAP, as for these databases vaccination information was retrieved through linkage with vaccination registries. For all other data sources, exposure to vaccines was incomplete because vaccine exposure information was not embedded in the system. For all databases the presence of a vaccine record was assumed to equate to a vaccine administration.

### Study populations

Within each database, we identified three non-exclusive cohorts (Baden et al., 2021): The *post-vaccine period cohort* included all adults (age ≥18) with a recording of an event of interest, defined as either a venous thromboembolism (VTE), arterial thromboembolism (ATE), or thrombosis with thrombocytopenia syndrome (TTS) from December 2020 onwards (Polack et al., 2020). The *vaccinated cohort* comprised all adults with a VTE, ATE, or TTS recorded between 0 and 28 days after a dose of any COVID-19 vaccine. This follow-up period was selected based on the assumption that time-to-onset of TTS, ATE and VTE would be short. Finally, a (Voysey et al., 2021) *pre-vaccination (background) cohort* comprised all adults with a record of VTE, ATE, or TTS between January 2017 and November 2020.

The respective anchoring events were defined as follows: *TTS* were defined as a composite outcome of any thromboembolic event (TE) [VTE or ATE] in addition to thrombocytopenia diagnosis or a measurement of <150 × 10^9^/L platelets within 10 days of the TE event date. *VTE* were defined as a composite of deep vein thrombosis (DVT) and pulmonary embolism (PE). Finally, *ATE* was defined as a composite of ischemic stroke, myocardial infarction, and other rare thromboembolisms.

Participants in all three cohorts were required to have at least 1 year observation time in the respective database prior to the event of interest and at least 1 day of follow-up. Participants in the cohorts were followed up from the time of the respective TTS, VTE, or ATE event until the last available date in the respective database (i.e., until they were transferred out of the database, death, or the end of data collection, whichever occurred first). Detailed definitions for COVID-19 vaccine exposure and TTS, VTE, and ATE are included in Supplementary Material SA, SB.

### Treatments of interest

We investigated utilization of medicines, drug classes, and procedures as described in Table 2. Exposures to these drugs were identified based on the WHO’s Anatomical Therapeutic Chemical (ATC) classification, and utilization was assessed from the time of the TTS, VTE, or ATE diagnosis up to the last data available in each database. A code list is included in Supplementary Material SB.

**TABLE 2:** Drug classes, medicines and procedures.

Drug classes	Therapeutic subgroup	Ingredient-level subgroup	ATC code/ATC category
Systemic corticosteroids			H02AB
Anti-thrombotic treatments	Vitamin K antagonists		B01AA
	Heparins		B01AB
	Platelet aggregation inhibitors		B01AC
	Thrombolytic/fibrinolytic enzymes		B01AD
	Thrombolytic procedures (i.e., catheter-directed thrombolysis)		procedures
	Direct thrombin inhibitors	Dabigatran	B01AE07
		Direct thrombin inhibitors excl. dabigatran	B01AE
	Direct factor Xa inhibitors		B01AF
	Other anticoagulants		B01AX
Rituximab			L01XC02
Fibrinogen			B02BB
Immunoglobulins			J06B
Plasma exchange/platelet transfusion			procedures, B05AX03

### Treatment pathways

For all patients in each cohort, we investigated treatment pathways, defined as the sequence of received medications and/or procedures over time. The R package “TreatmentPatterns” was used to construct treatment pathways from a person’s medical history (Markus et al., 2022). We defined treatments as continuous sequences of exposure records from the same treatment with a maximum gap of 7 days between exposures. All treatment episodes after the diagnosis of TTS, VTE, or ATE were included, regardless of their duration to allow for the study of one-off infusions such as plasma or intravenous glucocorticoids. If a patient received different treatments at the same time for at least 1 day, this was considered as combination therapy. The full study settings are included in Supplementary Material SC. For further details on the construction of treatment pathways we refer the interested reader to earlier work (Markus et al., 2022).

### Statistical analyses

We stratified each of the cohorts according to diagnosis (TTS, VTE, or ATE) for our analyses. Descriptive statistics were provided to characterize the respective cohorts for each database. To visualize treatment patterns over time, we summarized patient’s treatment pathways in sunburst plots, which show the first treatments in the centre and subsequent treatments in the surrounding outer layers. Combination therapy is depicted as a sliced portion with two different colours. Cell counts less than 5 were suppressed as required by most databases for privacy protection. These individual treatment patterns were aggregated into one slice called “Other”, which is depicted in grey in the sunburst plots. For full transparency and reproducibility, the code needed to run the analysis on a database mapped to OMOP CDM is available at: https://github.com/oxford-pharmacoepi/TreatmentPatternsDUS.

## Results

All results are available online in an interactive web application: https://dpa-pde-oxford.shinyapps.io/ROC22_TreatmentPatterns/. The most relevant information and figures are summarized below. Results for the post-vaccine period population cohort are included in Supplementary Material SD.

### Treatment pathways in patients with TTS

The number of TTS cases in each cohort and selected baseline characteristics for each database are detailed in Table 3. A large difference in the proportion of people with background TTS (in the pre-vaccination period) treated with any medicine of interest was seen between databases, ranging from 49.7% (Hospital CDM) to 82.8% (IMASIS) for inpatient and 6.9% (CPRD) to 71.2% (LPD France) for outpatient databases (Table 3). The proportions of people treated following TTS after vaccination were similarly heterogeneous.

**TABLE 3:** Baseline characteristics of pre-vaccination *versus* vaccinated TTS patients.

	Characteristic	Hospital CDM (United States)	IMASIS (ES)	CPRD (United Kingdom)	SIDIAP (ES)	IPCI (NL)	LPD France (FR)	DA Germany (DE)	Open Claims (United States)
**Pre-vaccination cohort**	Number of patients	40,252	829	1,720	8,425	92	73	971	208,190
	Treated, %	49.7	82.8	6.9	38.1	23.9	71.2	20.9	8.8
	Follow up time after index (days), Mean	281.8	464	629.6	584.7	728.1	759.7	676.3	629.1
	Gender: Male, %	57.3	62.2	70.9	68.0	67.4	69.9	67.6	56.8
	Age at index (years), Mean (SD)	66.1 (13.0)	73.0 (14.1)	71.1 (13.7)	73.2 (13.5)	70.1 (17.1)	70.3 (16.0)	71.9 (13.5)	67.1 (13.9)
	Charlson comorbidity index, Mean (SD)	6.4 (3.7)	3.5 (3.2)	3.1 (2.4)	5.0 (3.4)	2.7 (2.0)	1.9 (1.8)	2.7 (3.4)	6.4 (3.9)
**Vaccinated cohort**	Number of patients	43	5	50	258	N/A	N/A	8	1,220
	Treated, %	41.9	100.0	6.0	33.7	N/A	N/A	12.5	9.3
	Follow up time after index (days), Mean	53.2	101.4	51.2	56.4	N/A	N/A	89.8	150.9
	Gender: Male, %	55.8	0.0	66.0	70.9	N/A	N/A	62.5	56.6
	Age at index (years), Mean (SD)	68.9 (11.0)	61.8 (11.8)	69.7 (18.1)	76.2 (11.7)	N/A	N/A	67.9 (17.1)	68.7 (13.1)
	Charlson comorbidity index, Mean (SD)	7.0 (4.3)	4.2 (3.0)	2.9 (2.2)	5.1 (3.3)	N/A	N/A	5.8 (2.8)	6.8 (4.0)

N/A—Not available, cell count <5.

#### Pre-vaccination cohort

**FIGURE 1:**
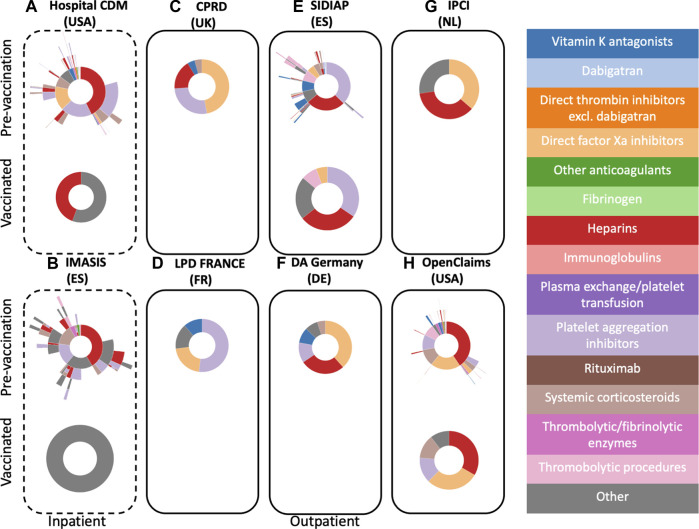
Sunburst plots visualizing treatment pathways for TTS patients in pre-vaccination cohorts (top) *versus* vaccinated cohorts (bottom). Inpatient databases are depicted with a dashed line frame, whilst outpatient ones have a solid frame.

Figure 1 illustrates the treatment patterns following pre-vaccination TTS (recorded before 12/2020) and following COVID-19 vaccination. In the two hospital databases with inpatient records, 49.7% (Hospital CDM) and 82.8% (IMASIS) of people with a pre-vaccination TTS event received treatment with at least one of the medicines of interest. In Hospital CDM data, heparins, platelet aggregation inhibitors, and direct Xa inhibitors were frequent first treatments, used by 42.8%, 20.1%, and 15.5% of the treated TTS patients respectively (Figure 1A). In total 41.2% of TTS patients in Hospital CDM switched to another treatment of interest during the study period. Among pre-vaccination TTS patients receiving any of the treatments of interest in IMASIS (inpatient data), 41.7% had their first treatment with heparin for an average duration of approximately 5 days. Among these, 51.4% did not switch to another treatment during the study period. A further 16.2% and 14.5% of patients had platelet aggregation inhibitors and systemic corticosteroids as their first treatments respectively. Only 3.8% of these patients initiated treatment with thrombolytic enzymes (Figure 1B).

For outpatient therapies, proportions of TTS patients receiving any of the medicines of interest varied greatly between databases and setting, ranging from 6.9% in CPRD to 71.2% in France LPD (Table 3). Direct Xa inhibitors were most common as first treatment in patients with pre-vaccination TTS observed in CPRD, Germany DA, and IPCI. For SIDIAP and France LPD platelet aggregation inhibitors were the most frequently prescribed first-line treatments. Heparin was the most used first treatment in Open Claims (40.6%) and the second most used first treatment in SIDIAP, Germany DA, and IPCI. A small proportion of patients across databases initiated treatment with vitamin K antagonists. Most patients did not switch to a second treatment during the study period, but more treatments were prescribed to pre-vaccination TTS patients in SIDIAP and Open Claims (15.5% and 13.5% of patients received a follow-up treatment respectively).

#### Vaccinated cohort

Figure 1 also illustrates treatment patterns following TTS after vaccination. In the inpatient databases (Hospital CDM and IMASIS), only 43 and 5 patients had TTS after vaccination, respectively. Although all patients in IMASIS were treated, limited sample size did not allow to characterise these for privacy protection. Heparin was the most frequently used therapy in Hospital CDM (Figure 1A). No sunburst plots are available for TTS patients after vaccination in CPRD/Germany DA (less than 5 treated patients) and France LPD/IPCI (less than 5 patients).

In SIDIAP and Open Claims, heparins and platelet aggregation inhibitors were the most common first treatments (Figures 1E, H) with an average duration of approximately a month (SIDIAP, platelet aggregation inhibitors) and 2 days (Open Claims, heparins), respectively. Platelet aggregation inhibitors, direct Xa inhibitors, thrombolytic therapies, and systemic corticosteroids were also used in these patients in both databases. Treatment patterns in the post-vaccine period for TTS were similar to treatments of pre-vaccination period events for databases with sufficient sample size.

### Treatment pathways in patients with VTE and ATE

Similar to TTS, great variability was observed across databases in the proportion of people with VTE and ATE treated with at least one of the medicines of interest (Table 4). The proportion receiving any of the medicines under study ranged from 28.1% (Hospital CDM) to 64.4% (IMASIS) for inpatient and 12.5% (CPRD) to 53.1% (SIDIAP) for outpatient databases following a pre-vaccination VTE, and 28.5% (Hospital CDM) to 60.6% (IMASIS) for inpatient and 4.7% (CPRD) to 41.1% (SIDIAP) for outpatient databases following a pre-vaccination ATE. For vaccinated patients the ranges were similar; 9.5% (CPRD) to 70% (IMASIS) for VTE and 3.7% (CPRD) to 55.9% (France LPD) for ATE.

**TABLE 4:** Baseline characteristics of pre-vaccination *versus* vaccinated VTE and ATE patients.

		Characteristic	Hospital CDM (United States)	IMASIS (ES)	CPRD (United Kingdom)	SIDIAP (ES)	IPCI (NL)	LPD France (FR)	DA Germany (DE)	Open Claims (United States)
**Pre-vaccination cohort**	VTE	Number of patients	445,546	1,455	131,184	32,925	24,071	43,036	96,939	8,133,455
		Treated, %	28.1	64.4	12.5	53.1	22.0	27.8	22.9	20.8
		Follow up time after index (days), Mean	469.9	529.7	706	688.7	792.2	739.9	747.9	868.7
		Gender: Male, %	48.0	50.2	50.0	49.4	45.2	47.7	45.5	47.9
		Age at index (years), Mean (SD)	62.4 (12.1)	68.7 (14.2)	64.2 (13.3)	68.6 (13.9)	62.5 (11.9)	70.7 (12.4)	67.8 (13.0)	64.2 (12.5)
		Charlson comorbidity index, Mean (SD)	3.8 (3.3)	2.5 (2.7)	1.9 (2.0)	3.3 (2.9)	1.5 (1.7)	1.0 (1.3)	2.8 (2.8)	4.2 (3.5)
	ATE	Number of patients	859,044	4,364	195,692	95,081	117,798	221,716	193,918	25,780,080
		Treated, %	28.5	60.6	4.7	41.1	6.8	22.0	9.4	6.9
		Follow up time after index (days), Mean	420.8	550.9	765.1	715.9	775.6	769.8	792.3	775.4
		Gender: Male, %	54.7	58.1	67.4	58.6	66.2	73.6	62.2	51.1
		Age at index (years), Mean (SD)	66.4 (14.5)	72.2 (16.4)	68.0 (16.9)	73.1 (16.2)	69.2 (16.0)	68.8 (14.2)	70.1 (15.5)	69.3 (15.0)
		Charlson comorbidity index, Mean (SD)	4.7 (3.5)	2.6 (3.2)	2.7 (2.)	3.9 (3.2)	2.5 (1.5)	1.8 (1.4)	3.8 (2.9)	5.7 (3.5)
**Vaccinated cohort**	VTE	Number of patients	784	10	3,800	1,051	346	140	724	81,150
		Treated, %	16.8	70.0	9.5	45.2	25.1	60	28.6	23.4
		Follow up time after index (days), Mean	71.3	56.2	58.3	56.3	44.3	128.1	113	175.3
		Gender: Male, %	49.6	50.0	49.2	46.9	46	62.1	49.7	48.3
		Age at index (years), Mean (SD)	66.8 (14.4)	60.8 (10.4)	68.1 (15.6)	73.3 (13.8)	66.9 (11.2)	72.9 (10.5)	68.3 (13.2)	65.5 (14.4)
		Charlson comorbidity index, Mean (SD)	4.1 (3.6)	3.4 (2.8)	2.1 (2.2)	3.4 (3.1)	1.6 (1.6)	1.4 (1.2)	3.5 (3.1)	4.7 (3.6)
	ATE	Number of patients	1,334	21	3,841	2,820	1,408	639	1,446	197,811
		Treated, %	17.5	47.6	3.7	32.8	9.4	55.9	10.8	7.6
		Follow up time after index (days), Mean	68.5	68	58.3	54	44.3	139.1	123.3	174.9
		Gender: Male, %	55.0	66.7	65.7	59.3	67.5	76.1	67.4	51.8
		Age at index (years), Mean (SD)	68.1 (11.0)	66.7 (10.3)	70.2 (12.8)	75.2 (12.4)	69.7 (10.1)	67.8 (10.2)	68.1 (11.4)	69.3 (12.4)
		Charlson comorbidity index, Mean (SD)	4.7 (3.3)	5.5 (4.0)	2.7 (2.1)	3.9 (2.9)	2.6 (1.7)	2.1 (1.6)	4.0 (2.8)	6.2 (3.6)

#### Pre-vaccination cohort

**FIGURE 2:**
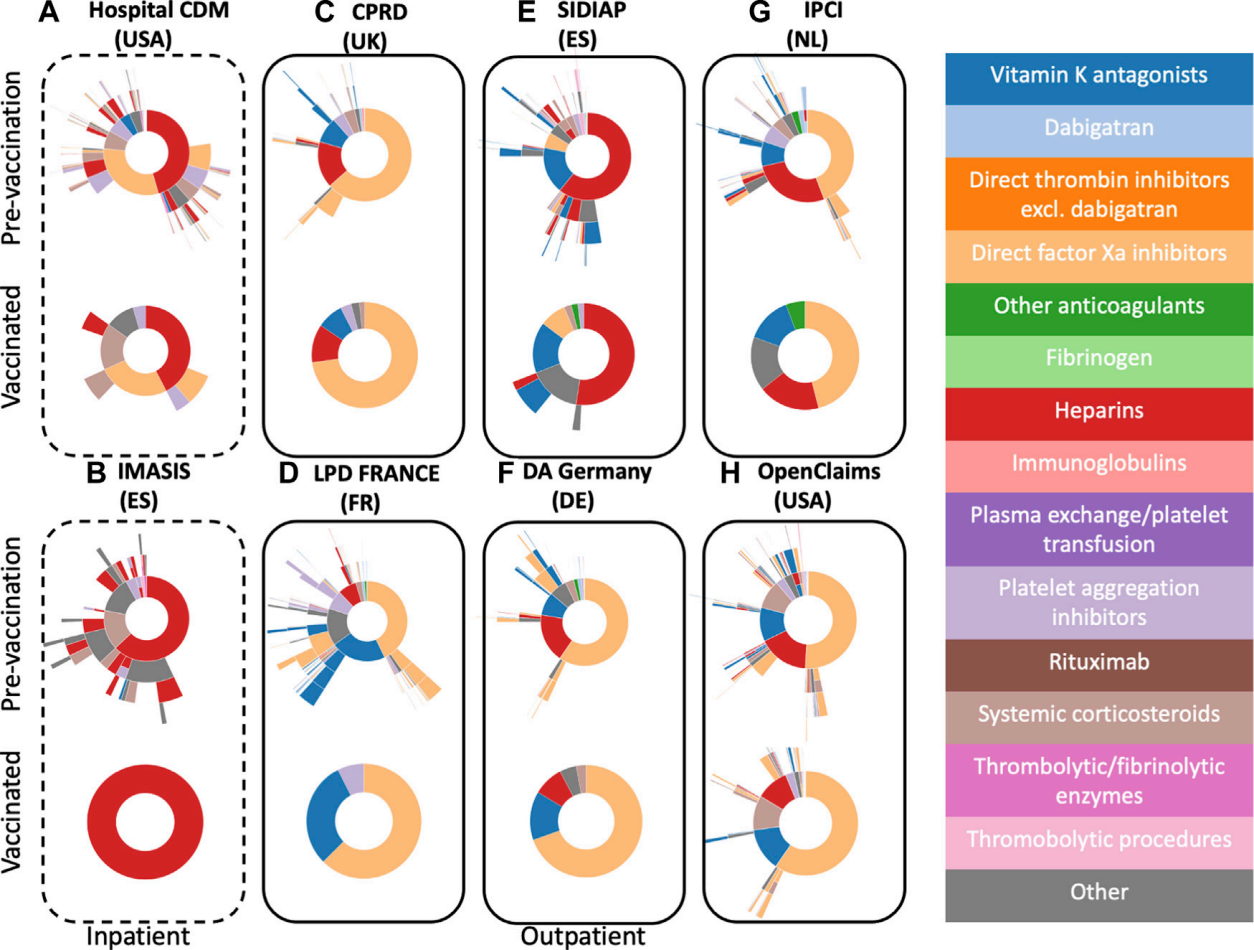
Sunburst plots visualizing treatment pathways for VTE patients in pre-vaccination cohorts (top) *versus* vaccinated cohorts (bottom). Inpatient databases are depicted with a dashed line frame, whilst outpatient ones have a solid frame.

Figure 2 illustrates the treatment patterns following pre-vaccination VTE (recorded before 12/2020) and following COVID-19 vaccination. Regarding inpatient therapies, 93.9% of the patients treated following pre-vaccination VTE received monotherapy, with heparins and direct Xa inhibitors being the two most common treatments. Those started with heparin were most likely to switch to direct Xa inhibitors and *vice versa*. About 50% of treated patients did not receive subsequent therapies. In the other inpatient database (IMASIS), heparins were the most used first treatment (64.8%) followed by systemic corticosteroids (15.6%). Almost all outpatient databases showed a majority of background VTE patients firstly treated with direct Xa inhibitors. An exception was SIDIAP, where heparins are the most first-line treatment (56.8%). In the other databases heparins were also seen as first treatment. Another common first treatment alternative was vitamin K antagonists (SIDIAP, LPD France). Subsequent treatments following a pre-vaccination VTE event were heterogeneous in our treatment pattern analysis.

**FIGURE 3:**
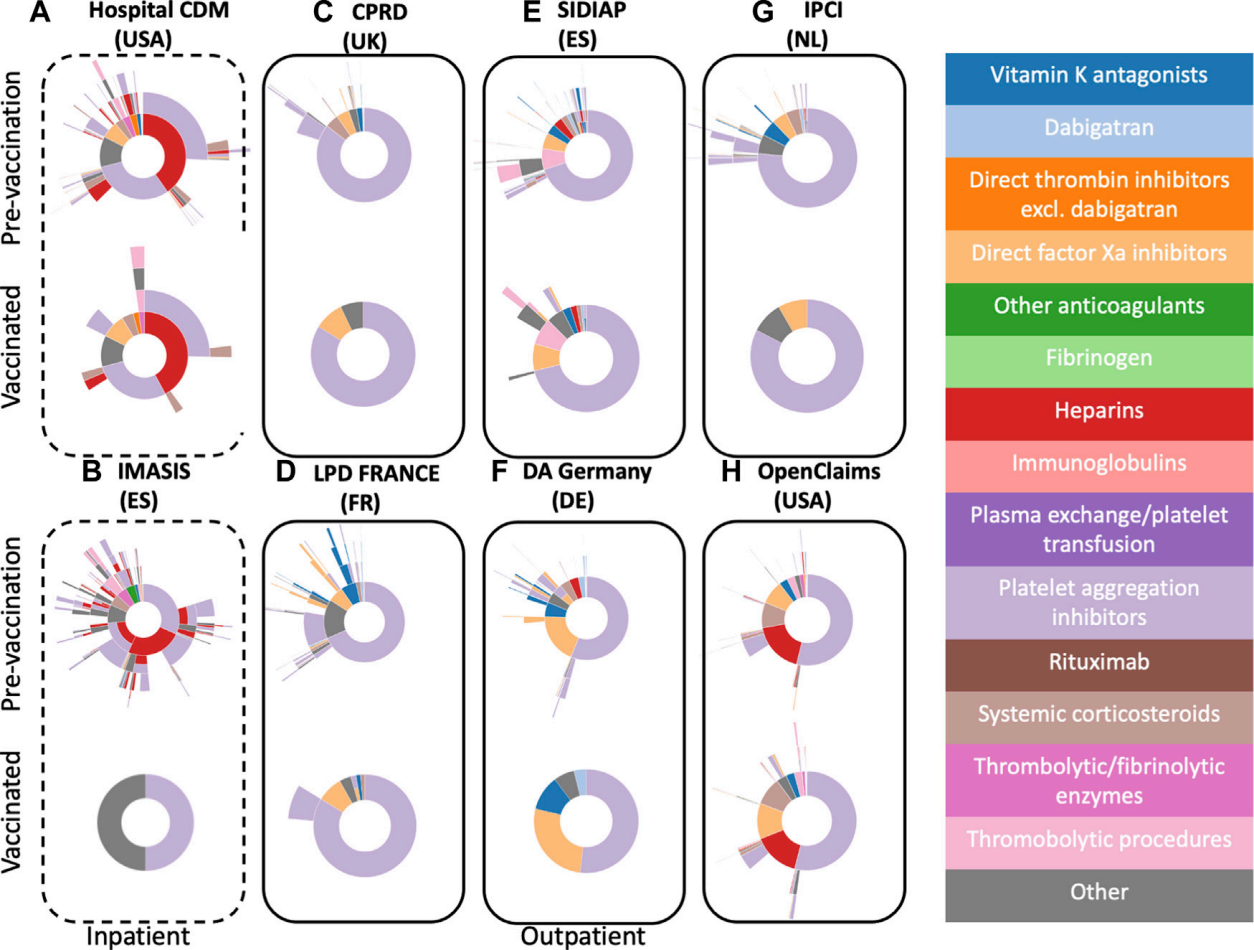
Sunburst plots visualizing treatment pathways for ATE patients in pre-vaccination cohorts (top) *versus* vaccinated cohorts (bottom). Inpatient databases are depicted with a dashed line frame, whilst outpatient ones have a solid frame.

Similarly, Figure 3 illustrates the treatment patterns following pre-vaccination ATE and following COVID-19 vaccination. Almost 75% of the ATE patients treated in hospital were administered heparins or platelet aggregation inhibitors as first treatments, either in monotherapy or in combination. Of those who started on heparins in monotherapy, approximately 60% of patients subsequently received platelet aggregation inhibitors as monotherapy or a combination therapy in IMASIS and Hospital CDM.

Likewise, platelet aggregation inhibitors were the most common first outpatient treatments in all contributing outpatient databases. Other less frequently used therapies included direct Xa inhibitors, vitamin K antagonists, thrombolytic therapies, and heparins.

#### Vaccinated cohort

Treatment patterns in vaccinated patients with VTE and ATE were very similar to those seen in pre-vaccination VTE and ATE patients respectively (see top *versus* bottom Figures 2, 3). In the inpatient databases, heparins were the most used treatment for VTE (note in IMASIS this is the only treatment that exceeds the minimum cell count of 5). Regarding outpatient treatments, direct Xa inhibitors were the most frequently prescribed first treatments for VTE in most of the contributing databases except for SIDIAP, where heparins (52.4%) and vitamin K antagonists (16.2%) were the most two commonly used therapies. Treatments used following ATE after vaccination were also similar, with heparins (Hospital CDM) and platelet aggregation inhibitors (all other databases) being the most common treatments (Figure 3). Smaller numbers of events meant less subsequent treatments were observed following vaccination as compared to pre-vaccination VTE and ATE. Treatment patterns in the post-vaccine period were comparable to treatments following ATE and VTE after vaccination.

## Discussion

To our knowledge, this is the first study characterizing treatment pathways in patients with TTS or VTE/ATE following COVID-19 vaccination. Our results suggest that drug utilization following VTE and ATE is more homogeneous compared to TTS, potentially due to the challenges associated with the management of the latter. Patterns of medical treatments for TTS, VTE, and ATE after vaccination were broadly similar to those seen for pre-vaccination events. The use of heparins following TTS in the post vaccination period suggests that clinicians did not classify these as vaccine-induced thrombosis with thrombocytopenia (VITT).

### Treatment patterns in patients with TTS

As TTS are particularly challenging to manage in clinical practice (Pavord et al., 2021), various guidelines on the treatment of thrombosis and TTS after vaccination have recently been issued. German and international guidelines (Nazy et al., 2021; Oldenburg et al., 2021) suggested to screen TTS patients for VITT using an ELISA immunoassay to detect anti-PF4 antibodies. For patients testing positive, heparins should be avoided, and in case of severe thromboembolic complications, intravenous (IV) immunoglobulins were recommended together with HIT-compatible anticoagulants such as danaparoid, argatroban, direct oral anticoagulants, or possibly fondaparinux. Italian (Gresele et al., 2021) and United Kingdom NICE guidelines (Pavord et al., 2021) made broadly similar recommendations, with additional mentioning other therapies including systemic corticosteroids in patients with low platelet counts (Gresele et al., 2021; Pavord et al., 2021), rituximab in patients with insufficient response to IV immunoglobulins (Gresele et al., 2021; Pavord et al., 2021), and platelet transfusion, which should be restricted to people with VITT with high bleeding risk requiring surgery (Pavord et al., 2021), severe thrombocytopenia or serious bleeding (Gresele et al., 2021). A recent publication from the US(14) updated diagnostic criteria for VITT and added more nuanced recommendations for its pharmacological management, including a preference for direct Xa inhibitors over vitamin K antagonists.

Before COVID-19 vaccines were available, most patients in hospital with TTS received treatment with heparins, platelet aggregation inhibitors, direct Xa inhibitors, or systemic glucocorticoids, either in monotherapy or in combination. As TTS events after vaccination recorded in hospital were very rare in our study, a detailed analysis of treatments was not possible due to information governance rules. TTS cases in the outpatient setting after vaccination were only available from Spanish primary care records (SIDIAP) and claims data from the US. In SIDIAP, platelet aggregation inhibitors were dispensed most frequently, followed by heparins, thrombolytic procedures, and direct Xa inhibitors, whereas results from Open Claims showed that most TTS cases after vaccination were treated with heparins, direct Xa inhibitors, and systemic glucocorticoids.

Information on measurement of anti-PF4 antibodies, which is required to reach a diagnosis of VITT, was not available in either of both datasets. We can therefore only assume that patients, for whom TTS after vaccination was treated with heparins despite guidelines contraindicating this, tested negative for anti-PF4 and were thus classified as TTS without VITT. Treatment guidelines for VITT only became available some time into the rollout of the vaccines, and hence were not available to clinicians at the start of the vaccination campaigns.

### Treatment patterns in patients with VTE and ATE

Compared to TTS, VTE without concurrent thrombocytopenia was common. Patients with VTE following vaccination were treated similarly compared to pre-vaccination VTE cases. This finding is in line with guidelines on the treatment of VTE in COVID-19 patients, which suggested the use of established treatment regimes, including low-molecule weight heparins in the inpatient and direct oral anticoagulants in the post-discharge setting (Kaptein et al., 2021).

Treatment pattern for ATE, a composite outcome of myocardial infarction, ischemic stroke, and other rare arterial thromboembolisms, were also broadly similar for pre-vaccination and after vaccination events. Platelet aggregation inhibitors were prescribed predominantly, and inpatient and claims data also showed substantial heparin use. Thrombolytic procedures were recorded in some databases. This is in line with pre-pandemic European Guidelines (Ibanez et al., 2017) on the management of acute myocardial infarction, highlighting anticoagulants (e.g., unfractionated heparin, enoxaparin) and dual antiplatelet therapy (low dose aspirin and prasugrel/ticagrelor or clopidogrel) as the cornerstone of pharmacotherapy accompanying primary percutaneous coronary interventions or fibrinolysis in the acute phase of STEMI. Dual antiplatelet therapy was also recommended for maintenance therapy, which is reflected in our study by the high proportion of antiplatelet therapies prescribed in outpatient databases following ATE. Heterogeneity in observed treatments can be due to different diagnosis and treatment protocols but also reflective of different reimbursement policies across the contributing countries.

For the clinical management of strokes associated with COVID-19 vaccination, the WHO recommends to follow the standard stroke protocols, including systemic thrombolysis and/or intraarterial thrombectomy, if required.

## Limitations

Our study is based on routinely collected healthcare data, with many of them being used for vaccine safety studies in the context of the pandemic. We are aware that previous research showed heterogeneity in incidence estimates of thromboembolic events across databases: particularly for thrombocytopenia, results vary considerably depending on the degree to which platelet measurements are captured (Burn et al., 2022a; Burn et al., 2022b). Therefore, some degree of measurement error is expected. Case reports indicated that the location of thrombosis after vaccination has been atypical, with CVST being reported more frequently. Despite based on Brighton criteria, TTS in our data cannot be equated to VITT, as we did not have access to highly specific antibodies (anti-PF4) required to reach a VITT diagnosis. A lack of definite VITT classification could explain why a proportion of the observed people with TTS following vaccination were still treated with heparins despite guidelines advising against this. We expect that those patients were indeed tested, but negative results for anti-PF4 antibodies were retrieved and events were subsequently classified as TTS without VITT.

TE are serious events often requiring hospital treatment. “First” treatments identified from the outpatient setting may therefore present subsequent treatments (after discharge) rather than initial therapy. Additionally, we cannot rule out some degree of missingness of recorded prescriptions/dispensations, particularly as inpatient medication was not available for outpatient databases. Lastly, limited sample size meant that we could not report treatment patterns in patients with venous or arterial TTS separately as information government rules required suppression of cell counts less than 5 in most databases.

## Conclusion

The management of TTS, VTE, and ATE after vaccination appeared similar to that seen in pre-vaccination events. More data is needed on the long-term treatment and prognosis of TTS and VITT.

## Data Availability

The original contributions presented in the study are included in the article/Supplementary Material, further inquiries can be directed to the corresponding author.
