# Supplementary material for: Characterising the treatment of thromboembolic events after COVID-19 vaccination in 4 European countries and the US: An international network cohort study
Source: Front Pharmacol. 2023 Mar 24;14:1118203. doi: 10.3389/fphar.2023.1118203 (PMC10079887; doi:10.3389/fphar.2023.1118203)
Supplement: Supplementary file 1 [file DataSheet1.docx]

**SUPPLEMENTARY MATERIAL**

**Characterising the treatment of thromboembolic events after COVID-19 vaccination in 4 European countries and the US: an international network cohort study**

**SUPPLEMENTARY MATERIAL A: DEFINITIONS STUDY POPULATIONS**

**Cohort definition of vaccinated cohorts**

1. **TTS vaccinated cohort**

Initial Event Cohort:

People with continuous observation of 365 days before event may enter the cohort when observing any of the following:

- Condition occurrence of any thromboembolic event (TE).

Inclusion Criteria:

- Age greater than or equal to 18 years AND,

- Thrombocytopenia as defined by

- having at least 1 condition occurrence of Thrombocytopenia OR,

- having at least 1 measurement of Platelet measurement between 10-150 ("thousand per microliter", "thousand per cubic millimeter", "per cubic millimeter", "cubic millimeter", "billion per liter", "per liter" or "million per milliliter") OR,

- having at least 1 measurement of Platelet measurement between 10,000 -150,000 ("per microliter")

between 10 days before and 10 days after cohort entry start date AND,

- Having at least 1 COVID-19 vaccination between 28 days before and 0 days before cohort entry start date AND,

- No condition occurrences of any TE in the 365 days prior to cohort entry date AND,

- Index date after November 30, 2020.

Cohort Exit: the cohort end date will be offset from index event's end date plus 1 day.

1. **VTE vaccinated cohort**

Initial Event Cohort:

People with continuous observation of 365 days before event may enter the cohort when observing any of the following:

- Condition occurrence of any deep vein thrombosis (DVT) or pulmonary embolism (PE).

Inclusion Criteria:

- Age greater than or equal to 18 years AND,

- Having at least 1 COVID-19 vaccination between 28 days before and 0 days before cohort entry start date AND,

- No condition occurrences of any TE in the 365 days prior to cohort entry date AND,

- Index date after November 30, 2020.

Cohort Exit: the cohort end date will be offset from index event's end date plus 1 day.

1. **ATE vaccinated cohort**

Initial Event Cohort:

People with continuous observation of 365 days before event may enter the cohort when observing any of the following:

- Condition occurrence of ischemic stroke, myocardial infarction, or other rare arterial thromboembolisms.

Inclusion Criteria:

- Age greater than or equal to 18 years AND,

- Having at least 1 COVID-19 vaccination between 28 days before and 0 days before cohort entry start date AND,

- No condition occurrences of any TE in the 365 days prior to cohort entry date AND,

- Index date after November 30, 2020.

Cohort Exit: the cohort end date will be offset from index event's end date plus 1 day.

**Cohort definition of post-vaccine period cohorts:**

- Adapted from the above definitions:
  - Remove: “Having at least 1 COVID-19 vaccination between 28 days before and 0 days before cohort entry start date”.

**Cohort definition of pre-vaccination (background) cohorts:**

- Adapted from the above definitions:
  - Remove: “Having at least 1 COVID-19 vaccination between 28 days before and 0 days before cohort entry start date”.
  - Change: “Index date after December 31, 2016 and before November 30, 2020.”

**SUPPLEMENTARY MATERIAL B: CODE LIST**

**Thromboembolic events**

List of thromboembolic events (TE):

- *Deep vein thrombosis (DVT)*

| Concept ID | Concept name | Vocabulary |
| --- | --- | --- |
| 762047 | Acute bilateral thrombosis of subclavian veins | SNOMED |
| 762148 | Acute deep vein thrombosis of bilateral iliac veins | SNOMED |
| 761444 | Acute deep vein thrombosis of bilateral lower limbs following coronary artery bypass graft | SNOMED |
| 35616028 | Acute deep vein thrombosis of left iliac vein | SNOMED |
| 35615035 | Acute deep vein thrombosis of left lower limb following procedure | SNOMED |
| 761416 | Acute deep vein thrombosis of left upper limb following coronary artery bypass graft | SNOMED |
| 35615031 | Acute deep vein thrombosis of left upper limb following procedure | SNOMED |
| 43531681 | Acute deep vein thrombosis of lower limb | SNOMED |
| 35616027 | Acute deep vein thrombosis of right iliac vein | SNOMED |
| 35615034 | Acute deep vein thrombosis of right lower limb following procedure | SNOMED |
| 761415 | Acute deep vein thrombosis of right upper limb following coronary artery bypass graft | SNOMED |
| 35615030 | Acute deep vein thrombosis of right upper limb following procedure | SNOMED |
| 44782746 | Acute deep venous thrombosis | SNOMED |
| 44782751 | Acute deep venous thrombosis of axillary vein | SNOMED |
| 762008 | Acute deep venous thrombosis of bilateral axillary veins | SNOMED |
| 760875 | Acute deep venous thrombosis of bilateral calves | SNOMED |
| 765155 | Acute deep venous thrombosis of bilateral ileofemoral veins | SNOMED |
| 762017 | Acute deep venous thrombosis of bilateral internal jugular veins | SNOMED |
| 762417 | Acute deep venous thrombosis of bilateral legs | SNOMED |
| 762020 | Acute deep venous thrombosis of bilateral popliteal veins | SNOMED |
| 765546 | Acute deep venous thrombosis of bilateral tibial veins | SNOMED |
| 762004 | Acute deep venous thrombosis of both upper extremities | SNOMED |
| 44782742 | Acute deep venous thrombosis of calf | SNOMED |
| 44782747 | Acute deep venous thrombosis of femoral vein | SNOMED |
| 762015 | Acute deep venous thrombosis of ileofemoral vein of left leg | SNOMED |
| 765541 | Acute deep venous thrombosis of ileofemoral vein of right lower extremity | SNOMED |
| 44782748 | Acute deep venous thrombosis of iliofemoral vein | SNOMED |
| 44782752 | Acute deep venous thrombosis of internal jugular vein | SNOMED |
| 762009 | Acute deep venous thrombosis of left axillary vein | SNOMED |
| 760876 | Acute deep venous thrombosis of left calf | SNOMED |
| 765540 | Acute deep venous thrombosis of left femoral vein | SNOMED |
| 765922 | Acute deep venous thrombosis of left internal jugular vein | SNOMED |
| 762418 | Acute deep venous thrombosis of left lower extremity | SNOMED |
| 765537 | Acute deep venous thrombosis of left upper extremity | SNOMED |
| 44782767 | Acute deep venous thrombosis of lower extremity as complication of procedure | SNOMED |
| 46270071 | Acute deep venous thrombosis of lower limb due to coronary artery bypass grafting | SNOMED |
| 762022 | Acute deep venous thrombosis of politeal vein of right leg | SNOMED |
| 44782743 | Acute deep venous thrombosis of popliteal vein | SNOMED |
| 762021 | Acute deep venous thrombosis of popliteal vein of left leg | SNOMED |
| 762010 | Acute deep venous thrombosis of right axillary vein | SNOMED |
| 760877 | Acute deep venous thrombosis of right calf | SNOMED |
| 762013 | Acute deep venous thrombosis of right femoral vein | SNOMED |
| 762018 | Acute deep venous thrombosis of right internal jugular vein | SNOMED |
| 762419 | Acute deep venous thrombosis of right lower extremity | SNOMED |
| 762005 | Acute deep venous thrombosis of right upper extremity | SNOMED |
| 44782745 | Acute deep venous thrombosis of thigh | SNOMED |
| 44782744 | Acute deep venous thrombosis of tibial vein | SNOMED |
| 762026 | Acute deep venous thrombosis of tibial vein of left leg | SNOMED |
| 765156 | Acute deep venous thrombosis of tibial vein of right leg | SNOMED |
| 44782421 | Acute deep venous thrombosis of upper extremity | SNOMED |
| 764016 | Acute deep venous thrombosis of upper extremity after coronary artery bypass graft | SNOMED |
| 44782766 | Acute deep venous thrombosis of upper extremity as complication of procedure | SNOMED |
| 762048 | Acute thrombosis of left subclavian vein | SNOMED |
| 45757410 | Acute thrombosis of mesenteric vein | SNOMED |
| 762049 | Acute thrombosis of right subclavian vein | SNOMED |
| 36712892 | Acute thrombosis of splenic vein | SNOMED |
| 44782762 | Acute thrombosis of subclavian vein | SNOMED |
| 37109253 | Bilateral acute deep vein thrombosis of femoral veins | SNOMED |
| 40478951 | Bilateral deep vein thrombosis of lower extremities | SNOMED |
| 4046884 | Deep vein thrombosis of leg related to air travel | SNOMED |
| 4133004 | Deep venous thrombosis | SNOMED |
| 4181315 | Deep venous thrombosis associated with coronary artery bypass graft | SNOMED |
| 45773536 | Deep venous thrombosis of femoropopliteal vein | SNOMED |
| 763942 | Deep venous thrombosis of left lower extremity | SNOMED |
| 761980 | Deep venous thrombosis of left upper extremity | SNOMED |
| 443537 | Deep venous thrombosis of lower extremity | SNOMED |
| 4133975 | Deep venous thrombosis of pelvic vein | SNOMED |
| 40480555 | Deep venous thrombosis of peroneal vein | SNOMED |
| 4322565 | Deep venous thrombosis of profunda femoris vein | SNOMED |
| 763941 | Deep venous thrombosis of right lower extremity | SNOMED |
| 761928 | Deep venous thrombosis of right upper extremity | SNOMED |
| 4207899 | Deep venous thrombosis of tibial vein | SNOMED |
| 4028057 | Deep venous thrombosis of upper extremity | SNOMED |
| 193512 | Embolism and thrombosis of the renal vein | SNOMED |
| 435565 | Embolism and thrombosis of the vena cava | SNOMED |
| 4119760 | Iliofemoral deep vein thrombosis | SNOMED |
| 4124856 | Inferior mesenteric vein thrombosis | SNOMED |
| 4281689 | Phlegmasia alba dolens | SNOMED |
| 4284538 | Phlegmasia cerulea dolens | SNOMED |
| 4309333 | Postoperative deep vein thrombosis | SNOMED |
| 46285905 | Provoked deep vein thrombosis | SNOMED |
| 4033521 | Splenic vein thrombosis | SNOMED |
| 4055089 | Superior mesenteric vein thrombosis | SNOMED |
| 42538533 | Thrombosis of iliac vein | SNOMED |
| 44811347 | Thrombosis of internal jugular vein | SNOMED |
| 765049 | Thrombosis of left peroneal vein | SNOMED |
| 4317289 | Thrombosis of mesenteric vein | SNOMED |
| 4203836 | Thrombosis of subclavian vein | SNOMED |
| 4175649 | Thrombosis of the popliteal vein | SNOMED |
| 4153353 | Traumatic thrombosis of axillary vein | SNOMED |
| 46285904 | Unprovoked deep vein thrombosis | SNOMED |
| 4221821 | Thrombophlebitis of deep veins of lower extremity | SNOMED |
| 46271900 | Recurrent deep vein thrombosis | SNOMED |
| 4189004 | Deep vein thrombosis of leg related to intravenous drug use | SNOMED |

- *Pulmonary embolism (PE)*

| Concept ID | Concept name | Vocabulary |
| --- | --- | --- |
| 4120091 | Acute massive pulmonary embolism | SNOMED |
| 45768439 | Acute pulmonary embolism | SNOMED |
| 45768888 | Acute pulmonary thromboembolism | SNOMED |
| 4309039 | Hemorrhagic pulmonary infarction | SNOMED |
| 762808 | Infarction of lung due to embolus | SNOMED |
| 40480461 | Infarction of lung due to iatrogenic pulmonary embolism | SNOMED |
| 4108681 | Postoperative pulmonary embolus | SNOMED |
| 4091708 | Pulmonary air embolism | SNOMED |
| 440417 | Pulmonary embolism | SNOMED |
| 37109911 | Pulmonary embolism due to and following acute myocardial infarction | SNOMED |
| 37016922 | Pulmonary embolism on long-term anticoagulation therapy | SNOMED |
| 43530605 | Pulmonary embolism with pulmonary infarction | SNOMED |
| 4119608 | Pulmonary fat embolism | SNOMED |
| 254662 | Pulmonary infarction | SNOMED |
| 4253796 | Pulmonary microemboli | SNOMED |
| 45766471 | Pulmonary oil microembolism | SNOMED |
| 4121618 | Pulmonary thromboembolism | SNOMED |
| 4119610 | Pulmonary tumor embolism | SNOMED |
| 4119607 | Subacute massive pulmonary embolism | SNOMED |
| 4119609 | Subacute pulmonary fat embolism | SNOMED |
| 4236271 | Recurrent pulmonary embolism | SNOMED |

- *Venous thromboembolism (VTE) as a composite of DVT and PE*
- *Cerebral venous sinus thrombosis (CVST)*

| Concept ID | Concept name | Vocabulary |
| --- | --- | --- |
| 4102202 | Cerebral venous sinus thrombosis | SNOMED |
| 4048786 | Cerebral venous thrombosis of sigmoid sinus | SNOMED |
| 4043735 | Cerebral venous thrombosis of straight sinus | SNOMED |
| 4111713 | Non-pyogenic venous sinus thrombosis | SNOMED |
| 314667 | Nonpyogenic thrombosis of intracranial venous sinus | SNOMED |
| 4116206 | Septic thrombophlebitis of cavernous sinus | SNOMED |
| 4121335 | Septic thrombophlebitis of lateral sinus | SNOMED |
| 4119136 | Septic thrombophlebitis of sagittal sinus | SNOMED |
| 4041680 | Septic thrombophlebitis of sigmoid sinus | SNOMED |
| 4100225 | Thrombophlebitis lateral venous sinus | SNOMED |
| 4217471 | Thrombophlebitis of basilar sinus | SNOMED |
| 4104695 | Thrombophlebitis of cavernous sinus | SNOMED |
| 4167985 | Thrombophlebitis of inferior sagittal sinus | SNOMED |
| 764714 | Thrombophlebitis of sigmoid sinus | SNOMED |
| 4100224 | Thrombophlebitis of superior longitudinal venous sinus | SNOMED |
| 4098706 | Thrombophlebitis of superior sagittal sinus | SNOMED |
| 4277833 | Thrombophlebitis of torcular Herophili | SNOMED |
| 764710 | Thrombophlebitis of transverse sinus | SNOMED |
| 4228209 | Thrombosis of basilar sinus | SNOMED |
| 4234264 | Thrombosis of cavernous venous sinus | SNOMED |
| 4048890 | Thrombosis of inferior sagittal sinus | SNOMED |
| 4057329 | Thrombosis of lateral venous sinus | SNOMED |
| 4102203 | Thrombosis of superior longitudinal sinus | SNOMED |
| 4290940 | Thrombosis of superior sagittal sinus | SNOMED |
| 4079905 | Thrombosis of torcular Herophili | SNOMED |
| 4105338 | Thrombosis transverse sinus | SNOMED |

- *Splanchnic and visceral vein thrombosis (SVT)*
  - Splenic vein thrombosis

| Concept ID | Concept name | Vocabulary |
| --- | --- | --- |
| 4033521 | Splenic vein thrombosis | SNOMED |
| 36712892 | Acute thrombosis of splenic vein | SNOMED |

- - Splanchnic Vein Thrombosis

| Concept ID | Concept name | Vocabulary |
| --- | --- | --- |
| 4033521 | Splenic vein thrombosis | SNOMED |
| 196715 | Budd-Chiari syndrome | SNOMED |
| 199837 | Portal vein thrombosis | SNOMED |
| 4317289 | Thrombosis of mesenteric vein | SNOMED |
| 4092406 | Portal thrombophlebitis | SNOMED |
| 36712892 | Acute thrombosis of splenic vein | SNOMED |
| 4173167 | Mesenteric embolus | SNOMED |
| 4144032 | Mesenteric thrombus and/or embolus | SNOMED |
| 45757410 | Acute thrombosis of mesenteric vein | SNOMED |
| 45757409 | Chronic thrombosis of mesenteric vein | SNOMED |
| 4318407 | Thrombophlebitis of mesenteric vein | SNOMED |
| 4124856 | Inferior mesenteric vein thrombosis | SNOMED |
| 4055089 | Superior mesenteric vein thrombosis | SNOMED |

- - Portal vein thrombosis

| Concept ID | Concept name | Vocabulary |
| --- | --- | --- |
| 199837 | Portal vein thrombosis | SNOMED |

- - Mesenteric vein thrombosis

| Concept ID | Concept name | Vocabulary |
| --- | --- | --- |
| 36717492 | Acute occlusion of mesenteric vein | SNOMED |
| 45757410 | Acute thrombosis of mesenteric vein | SNOMED |
| 4124856 | Inferior mesenteric vein thrombosis | SNOMED |
| 4055089 | Superior mesenteric vein thrombosis | SNOMED |
| 4317289 | Thrombosis of mesenteric vein | SNOMED |
| 45757409 | Chronic thrombosis of mesenteric vein | SNOMED |
| 4318407 | Thrombophlebitis of mesenteric vein | SNOMED |
| 4173167 | Mesenteric embolus | SNOMED |
| 4144032 | Mesenteric thrombus and/or embolus | SNOMED |

- - Visceral venous thrombosis or obstruction

| Concept ID | Concept name | Vocabulary |
| --- | --- | --- |
| 36717492 | Acute occlusion of mesenteric vein | SNOMED |
| 36712892 | Acute thrombosis of splenic vein | SNOMED |
| 196715 | Budd-Chiari syndrome | SNOMED |
| 35624285 | Complete obstruction of hepatic portal vein | SNOMED |
| 4301208 | Hepatic vein thrombosis | SNOMED |
| 37110194 | Hepatic veno-occlusive disease with immunodeficiency syndrome | SNOMED |
| 37109927 | Obstruction of visceral vein | SNOMED |
| 4238060 | Portal vein obstruction | SNOMED |
| 4033521 | Splenic vein thrombosis | SNOMED |
| 4277276 | Veno-occlusive disease of the liver | SNOMED |
| 37111372 | Visceral venous thrombosis | SNOMED |
| 36712891 | Chronic thrombosis of splenic vein | SNOMED |

- *Ischemic stroke*

| Concept ID | Concept name | Vocabulary |
| --- | --- | --- |
| 4045735 | Anterior cerebral circulation infarction | SNOMED |
| 4031045 | Anterior choroidal artery syndrome | SNOMED |
| 761110 | Bilateral cerebral infarction due to precererbral arterial occlusion | SNOMED |
| 4110189 | Cerebral infarct due to thrombosis of precerebral arteries | SNOMED |
| 443454 | Cerebral infarction | SNOMED |
| 762951 | Cerebral infarction due to anterior cerebral artery occlusion | SNOMED |
| 765515 | Cerebral infarction due to basilar artery stenosis | SNOMED |
| 43530683 | Cerebral infarction due to carotid artery occlusion | SNOMED |
| 762933 | Cerebral infarction due to cerebral artery occlusion | SNOMED |
| 762937 | Cerebral infarction due to cerebral venous thrombosis | SNOMED |
| 4111714 | Cerebral infarction due to cerebral venous thrombosis, non-pyogenic | SNOMED |
| 4108356 | Cerebral infarction due to embolism of cerebral arteries | SNOMED |
| 45772786 | Cerebral infarction due to embolism of middle cerebral artery | SNOMED |
| 4110190 | Cerebral infarction due to embolism of precerebral arteries | SNOMED |
| 762935 | Cerebral infarction due to internal carotid artery occlusion | SNOMED |
| 763015 | Cerebral infarction due to middle cerebral artery occlusion | SNOMED |
| 46273649 | Cerebral infarction due to occlusion of basilar artery | SNOMED |
| 35610084 | Cerebral infarction due to occlusion of cerebral artery | SNOMED |
| 46270031 | Cerebral infarction due to occlusion of precerebral artery | SNOMED |
| 762934 | Cerebral infarction due to posterior cerebral artery occlusion | SNOMED |
| 43531607 | Cerebral infarction due to stenosis of carotid artery | SNOMED |
| 35610085 | Cerebral infarction due to stenosis of cerebral artery | SNOMED |
| 46270381 | Cerebral infarction due to stenosis of precerebral artery | SNOMED |
| 4110192 | Cerebral infarction due to thrombosis of cerebral arteries | SNOMED |
| 45767658 | Cerebral infarction due to thrombosis of middle cerebral artery | SNOMED |
| 44782773 | Cerebral infarction due to vertebral artery occlusion | SNOMED |
| 46270380 | Cerebral infarction due to vertebral artery stenosis | SNOMED |
| 37110678 | Cerebral ischemic stroke due to occlusion of extracranial large artery | SNOMED |
| 37110679 | Cerebral ischemic stroke due to stenosis of extracranial large artery | SNOMED |
| 4043731 | Infarction - precerebral | SNOMED |
| 4131383 | Infarction of basal ganglia | SNOMED |
| 4046237 | Infarction of optic radiation | SNOMED |
| 4119140 | Infarction of visual cortex | SNOMED |
| 4141405 | Left sided cerebral infarction | SNOMED |
| 37116473 | Multifocal cerebral infarction due to and following procedure on cardiovascular system | SNOMED |
| 4077086 | Occipital cerebral infarction | SNOMED |
| 4046359 | Partial anterior cerebral circulation infarction | SNOMED |
| 4319146 | Pituitary infarction | SNOMED |
| 4146185 | Right sided cerebral infarction | SNOMED |
| 36717605 | Silent cerebral infarct | SNOMED |
| 4142739 | Thalamic infarction | SNOMED |
| 4046358 | Total anterior cerebral circulation infarction | SNOMED |
| 372924 | Cerebral artery occlusion | SNOMED |

- *Myocardial infarction*

| Concept ID | Concept name | Vocabulary |
| --- | --- | --- |
| 4119457 | Acute Q wave infarction - anterolateral | SNOMED |
| 4119943 | Acute Q wave infarction - anteroseptal | SNOMED |
| 4121464 | Acute Q wave infarction - inferior | SNOMED |
| 4121465 | Acute Q wave infarction - inferolateral | SNOMED |
| 4124684 | Acute Q wave infarction - lateral | SNOMED |
| 4119948 | Acute Q wave infarction - widespread | SNOMED |
| 4126801 | Acute Q wave myocardial infarction | SNOMED |
| 4296653 | Acute ST segment elevation myocardial infarction | SNOMED |
| 46270162 | Acute ST segment elevation myocardial infarction due to left coronary artery occlusion | SNOMED |
| 761737 | Acute ST segment elevation myocardial infarction due to occlusion of circumflex coronary artery | SNOMED |
| 46270163 | Acute ST segment elevation myocardial infarction due to right coronary artery occlusion | SNOMED |
| 43020460 | Acute ST segment elevation myocardial infarction involving left anterior descending coronary artery | SNOMED |
| 45766076 | Acute ST segment elevation myocardial infarction of anterior wall involving right ventricle | SNOMED |
| 761736 | Acute ST segment elevation myocardial infarction of anteroapical wall | SNOMED |
| 46270159 | Acute ST segment elevation myocardial infarction of anterolateral wall | SNOMED |
| 46270160 | Acute ST segment elevation myocardial infarction of anteroseptal wall | SNOMED |
| 45766116 | Acute ST segment elevation myocardial infarction of inferior wall | SNOMED |
| 45766151 | Acute ST segment elevation myocardial infarction of inferior wall involving right ventricle | SNOMED |
| 35611570 | Acute ST segment elevation myocardial infarction of inferolateral wall | SNOMED |
| 35611571 | Acute ST segment elevation myocardial infarction of inferoposterior wall | SNOMED |
| 46274044 | Acute ST segment elevation myocardial infarction of lateral wall | SNOMED |
| 46270161 | Acute ST segment elevation myocardial infarction of posterior wall | SNOMED |
| 46273495 | Acute ST segment elevation myocardial infarction of posterobasal wall | SNOMED |
| 46270158 | Acute ST segment elevation myocardial infarction of posterolateral wall | SNOMED |
| 46270164 | Acute ST segment elevation myocardial infarction of septum | SNOMED |
| 45766075 | Acute anterior ST segment elevation myocardial infarction | SNOMED |
| 4178129 | Acute anteroapical myocardial infarction | SNOMED |
| 4267568 | Acute anteroseptal myocardial infarction | SNOMED |
| 312327 | Acute myocardial infarction | SNOMED |
| 44782769 | Acute myocardial infarction due to left coronary artery occlusion | SNOMED |
| 44782712 | Acute myocardial infarction due to right coronary artery occlusion | SNOMED |
| 45766115 | Acute myocardial infarction during procedure | SNOMED |
| 434376 | Acute myocardial infarction of anterior wall | SNOMED |
| 45766150 | Acute myocardial infarction of anterior wall involving right ventricle | SNOMED |
| 438438 | Acute myocardial infarction of anterolateral wall | SNOMED |
| 4243372 | Acute myocardial infarction of apical-lateral wall | SNOMED |
| 4108669 | Acute myocardial infarction of atrium | SNOMED |
| 4151046 | Acute myocardial infarction of basal-lateral wall | SNOMED |
| 4275436 | Acute myocardial infarction of high lateral wall | SNOMED |
| 438170 | Acute myocardial infarction of inferior wall | SNOMED |
| 45771322 | Acute myocardial infarction of inferior wall involving right ventricle | SNOMED |
| 438447 | Acute myocardial infarction of inferolateral wall | SNOMED |
| 441579 | Acute myocardial infarction of inferoposterior wall | SNOMED |
| 436706 | Acute myocardial infarction of lateral wall | SNOMED |
| 4324413 | Acute myocardial infarction of posterobasal wall | SNOMED |
| 4051874 | Acute myocardial infarction of posterolateral wall | SNOMED |
| 4303359 | Acute myocardial infarction of septum | SNOMED |
| 4147223 | Acute myocardial infarction with rupture of ventricle | SNOMED |
| 4145721 | Acute non-Q wave infarction | SNOMED |
| 4119944 | Acute non-Q wave infarction - anterolateral | SNOMED |
| 4119456 | Acute non-Q wave infarction - anteroseptal | SNOMED |
| 4119945 | Acute non-Q wave infarction - inferior | SNOMED |
| 4119946 | Acute non-Q wave infarction - inferolateral | SNOMED |
| 4121466 | Acute non-Q wave infarction - lateral | SNOMED |
| 4124685 | Acute non-Q wave infarction - widespread | SNOMED |
| 4270024 | Acute non-ST segment elevation myocardial infarction | SNOMED |
| 35610091 | Acute nontransmural myocardial infarction | SNOMED |
| 319039 | Acute posterior myocardial infarction | SNOMED |
| 444406 | Acute subendocardial infarction | SNOMED |
| 35610093 | Acute transmural myocardial infarction | SNOMED |
| 4119947 | Acute widespread myocardial infarction | SNOMED |
| 37109912 | Arrhythmia due to and following acute myocardial infarction | SNOMED |
| 438172 | Atrial septal defect due to and following acute myocardial infarction | SNOMED |
| 4124687 | Cardiac rupture due to and following acute myocardial infarction | SNOMED |
| 4215259 | First myocardial infarction | SNOMED |
| 4108678 | Hemopericardium due to and following acute myocardial infarction | SNOMED |
| 4173632 | Microinfarct of heart | SNOMED |
| 45771327 | Mitral valve regurgitation due to acute myocardial infarction with papillary muscle and chordal rupture | SNOMED |
| 45766214 | Mitral valve regurgitation due to acute myocardial infarction without papillary muscle and chordal rupture | SNOMED |
| 45766212 | Mitral valve regurgitation due to and following acute myocardial infarction | SNOMED |
| 4323202 | Mixed myocardial ischemia and infarction | SNOMED |
| 4329847 | Myocardial infarction | SNOMED |
| 37309626 | Myocardial infarction due to demand ischemia | SNOMED |
| 4170094 | Myocardial infarction in recovery phase | SNOMED |
| 4200113 | Non-Q wave myocardial infarction | SNOMED |
| 4030582 | Postoperative myocardial infarction | SNOMED |
| 35610087 | Postoperative nontransmural myocardial infarction | SNOMED |
| 4206867 | Postoperative subendocardial myocardial infarction | SNOMED |
| 35610089 | Postoperative transmural myocardial infarction | SNOMED |
| 4207921 | Postoperative transmural myocardial infarction of anterior wall | SNOMED |
| 4209541 | Postoperative transmural myocardial infarction of inferior wall | SNOMED |
| 37109911 | Pulmonary embolism due to and following acute myocardial infarction | SNOMED |
| 4108679 | Rupture of cardiac wall without hemopericardium as current complication following acute myocardial infarction | SNOMED |
| 4108219 | Rupture of chordae tendinae due to and following acute myocardial infarction | SNOMED |
| 4124686 | Silent myocardial infarction | SNOMED |
| 765132 | Subendocardial myocardial infarction | SNOMED |
| 45766114 | Subsequent ST segment elevation myocardial infarction | SNOMED |
| 45766113 | Subsequent ST segment elevation myocardial infarction of anterior wall | SNOMED |
| 45773170 | Subsequent ST segment elevation myocardial infarction of inferior wall | SNOMED |
| 4108217 | Subsequent myocardial infarction | SNOMED |
| 4108677 | Subsequent myocardial infarction of anterior wall | SNOMED |
| 4108218 | Subsequent myocardial infarction of inferior wall | SNOMED |
| 45766241 | Subsequent non-ST segment elevation myocardial infarction | SNOMED |
| 4108680 | Thrombosis of atrium, auricular appendage, and ventricle due to and following acute myocardial infarction | SNOMED |
| 439693 | True posterior myocardial infarction | SNOMED |
| 37109910 | Ventricular aneurysm due to and following acute myocardial infarction | SNOMED |

- *Other rare thromboembolisms*

| Concept ID | Concept name | Vocabulary |
| --- | --- | --- |
| 4195665 | Gastrointestinal tract vascular insufficiency | SNOMED |
| 4148299 | Ischemic colitis | SNOMED |
| 4173167 | Mesenteric embolus | SNOMED |
| 4317289 | Thrombosis of mesenteric vein | SNOMED |
| 4319280 | Acute bowel infarction | SNOMED |
| 4144032 | Mesenteric thrombus and/or embolus | SNOMED |
| 45757410 | Acute thrombosis of mesenteric vein | SNOMED |
| 45757409 | Chronic thrombosis of mesenteric vein | SNOMED |
| 44811741 | Acute ischaemia of large intestine | SNOMED |
| 44811740 | Acute ischaemia of small intestine | SNOMED |
| 37117790 | Insufficiency of mesenteric artery | SNOMED |
| 37016198 | Epiploic appendagitis | SNOMED |
| 35622081 | Nongangrenous ischemic colitis | SNOMED |
| 35622080 | Gangrenous ischemic colitis | SNOMED |
| 4345926 | Abdominal angina | SNOMED |
| 4342767 | Transient ischemic colitis | SNOMED |
| 4341648 | Hemorrhagic infarction of intestine | SNOMED |
| 4341646 | Occlusive mesenteric ischemia | SNOMED |
| 4340939 | Non-occlusive mesenteric ischemia | SNOMED |
| 4340378 | Transmural infarction of intestine | SNOMED |
| 4340375 | Focal segmental ischemia of small intestine | SNOMED |
| 4318537 | Large bowel gangrene | SNOMED |
| 4318407 | Thrombophlebitis of mesenteric vein | SNOMED |
| 4240850 | Acute ischemic enterocolitis | SNOMED |
| 4239942 | Embolic mesenteric infarction | SNOMED |
| 4237654 | Ischemic enterocolitis | SNOMED |
| 4215949 | Nonocclusive intestinal infarction | SNOMED |
| 4214720 | Thrombotic mesenteric infarction | SNOMED |
| 4192856 | Acute ischemic colitis | SNOMED |
| 4188336 | Chronic ischemic enterocolitis | SNOMED |
| 4174014 | Inferior mesenteric artery embolus | SNOMED |
| 4149013 | Mesenteric infarction | SNOMED |
| 4148257 | Chronic gastrointestinal tract vascular insufficiency | SNOMED |
| 4148256 | Acute GIT vascular insufficiency | SNOMED |
| 4124856 | Inferior mesenteric vein thrombosis | SNOMED |
| 4055089 | Superior mesenteric vein thrombosis | SNOMED |
| 4055025 | Superior mesenteric artery embolus | SNOMED |
| 4045408 | Ischemic stricture of intestine | SNOMED |
| 201894 | Acute vascular insufficiency of intestine | SNOMED |
| 192673 | Vascular insufficiency of intestine | SNOMED |

- *Arterial thromboembolism (ATE) as a composite of the three above*
- *Thrombocytopenia*
  - Platelet measurement

| Concept ID | Concept name | Vocabulary | Is excluded? | Include descendants? |
| --- | --- | --- | --- | --- |
| 3007461 | Platelets [#/volume] in Blood | LOINC | FALSE | TRUE |
| 3031586 | Platelets [#/volume] in Blood by Estimate | LOINC | FALSE | TRUE |
| 3024929 | Platelets [#/volume] in Blood by Automated count | LOINC | FALSE | TRUE |
| 3039827 | Platelets [#/volume] in Body fluid by Automated count | LOINC | FALSE | TRUE |
| 3024386 | Platelet mean volume [Entitic volume] in Blood by Rees-Ecker | LOINC | FALSE | TRUE |
| 4267147 | Platelet count | SNOMED | FALSE | TRUE |
| 37393863 | Platelet count | SNOMED | FALSE | TRUE |

- - Thrombocytopenia diagnosis

| Concept ID | Concept name | Vocabulary |
| --- | --- | --- |
| 37397537 | Beta thalassemia X-linked thrombocytopenia syndrome | SNOMED |
| 432870 | Thrombocytopenic disorder | SNOMED |
| 46272950 | Thrombocytopathy, asplenia and miosis | SNOMED |
| 44782445 | Thrombocytopenia due to alcohol | SNOMED |
| 42536958 | Pancytopenia caused by medication | SNOMED |
| 40321716 | Secondary thrombocytopenia | SNOMED |
| 37312165 | Atypical hemolytic uremic syndrome | SNOMED |
| 37209558 | Pancytopenia caused by immunosuppressant | SNOMED |
| 37204551 | Hereditary isolated aplastic anemia | SNOMED |
| 37204548 | Hereditary thrombocytopenia with normal platelets | SNOMED |
| 37204520 | Bleeding diathesis due to thromboxane synthesis deficiency | SNOMED |
| 37204478 | Pancytopenia due to IKZF1 mutations | SNOMED |
| 37117164 | Revesz syndrome | SNOMED |
| 37116398 | Thyrocerebrorenal syndrome | SNOMED |
| 37110394 | Isolated thrombocytopenia | SNOMED |
| 37019055 | Aplastic anemia co-occurrent with human immunodeficiency virus infection | SNOMED |
| 37018663 | Thrombocytopenia co-occurrent and due to alcoholism | SNOMED |
| 37017607 | Antibody mediated acquired pure red cell aplasia caused by erythropoiesis stimulating agent | SNOMED |
| 37017165 | GATA binding protein 1 related thrombocytopenia with dyserythropoiesis | SNOMED |
| 37016797 | MYH9 related disease | SNOMED |
| 37016151 | Aplastic anemia caused by antineoplastic agent | SNOMED |
| 36717326 | DK phocomelia syndrome | SNOMED |
| 36716406 | Severe fever with thrombocytopenia syndrome virus | SNOMED |
| 36716047 | Radioulnar synostosis with amegakaryocytic thrombocytopenia syndrome | SNOMED |
| 36715586 | Refractory thrombocytopenia | SNOMED |
| 36715053 | Autosomal dominant macrothrombocytopenia | SNOMED |
| 36713970 | WT limb blood syndrome | SNOMED |
| 36713443 | MYH9 macrothrombocytopenia syndrome | SNOMED |
| 36713112 | Pancytopenia due to antineoplastic chemotherapy | SNOMED |
| 36674972 | Macrothrombocytopenia with mitral valve insufficiency | SNOMED |
| 36674474 | Pancytopenia with developmental delay syndrome | SNOMED |
| 35625536 | Ataxia pancytopenia syndrome | SNOMED |
| 35623407 | Adult pure red cell aplasia | SNOMED |
| 4345236 | Parvoviral aplastic crisis | SNOMED |
| 4338386 | Thrombocytopenia due to non-immune destruction | SNOMED |
| 4316372 | HELLP syndrome | SNOMED |
| 4314802 | Kasabach-Merritt syndrome | SNOMED |
| 4311682 | Radial aplasia-thrombocytopenia syndrome | SNOMED |
| 4305588 | Doan-Wright syndrome | SNOMED |
| 4301602 | Thrombotic thrombocytopenic purpura | SNOMED |
| 4301128 | Thrombocytopenia due to diminished platelet production | SNOMED |
| 4300464 | Wiskott-Aldrich autosomal dominant variant syndrome | SNOMED |
| 4299560 | Thrombocytopenic purpura due to defective platelet production | SNOMED |
| 4298690 | Immunologic aplastic anemia | SNOMED |
| 4292531 | Thrombocytopenic purpura due to platelet consumption | SNOMED |
| 4292425 | Sex-linked thrombocytopenia | SNOMED |
| 4272928 | Thrombocytopenia due to hypersplenism | SNOMED |
| 4264464 | Mediterranean macrothrombocytopenia | SNOMED |
| 4258261 | Drug induced thrombotic thrombocytopenic purpura | SNOMED |
| 4247776 | Posttransfusion purpura | SNOMED |
| 4239484 | Acquired pancytopenia | SNOMED |
| 4235220 | Hereditary thrombocytopenic disorder | SNOMED |
| 4234973 | Chronic acquired pure red cell aplasia | SNOMED |
| 4233407 | Megakaryocytic aplasia | SNOMED |
| 4230266 | Autoimmune thrombotic thrombocytopenic purpura | SNOMED |
| 4226905 | Thrombocytopenia associated with AIDS | SNOMED |
| 4225810 | Aplastic anemia associated with AIDS | SNOMED |
| 4219476 | Thrombocytopenia due to defective platelet production | SNOMED |
| 4218171 | Uremic thrombocytopenia | SNOMED |
| 4214947 | Thrombocytopenic purpura associated with metabolic disorder | SNOMED |
| 4211348 | Aplastic anemia associated with pancreatitis | SNOMED |
| 4204900 | Acquired thrombotic thrombocytopenic purpura | SNOMED |
| 4197574 | Dilutional thrombocytopenia | SNOMED |
| 4188208 | Estren-Dameshek anemia | SNOMED |
| 4186108 | Aplastic anemia associated with metabolic alteration | SNOMED |
| 4185078 | Bernard Soulier syndrome | SNOMED |
| 4184758 | Acquired aplastic anemia | SNOMED |
| 4184200 | Secondary aplastic anemia | SNOMED |
| 4177177 | Cellular immunologic aplastic anemia | SNOMED |
| 4173278 | Thrombocytopenia due to blood loss | SNOMED |
| 4172008 | Cyclic thrombocytopenia | SNOMED |
| 4166754 | Perinatal thrombocytopenia | SNOMED |
| 4159966 | Upshaw-Schulman syndrome | SNOMED |
| 4159749 | Idiopathic maternal thrombocytopenia | SNOMED |
| 4159736 | Radiation thrombocytopenia | SNOMED |
| 4156233 | Thrombocytopenia due to sequestration | SNOMED |
| 4148471 | Fanconi’s anemia | SNOMED |
| 4147049 | Thrombocytopenia due to extracorporeal circulation | SNOMED |
| 4146088 | Aplastic anemia due to drugs | SNOMED |
| 4146086 | Constitutional aplastic anemia with malformation | SNOMED |
| 4145458 | Thrombocytopenia due to hypothermia | SNOMED |
| 4140545 | Post infectious thrombocytopenic purpura | SNOMED |
| 4139555 | Thrombocytopenia due to massive blood transfusion | SNOMED |
| 4137430 | Idiopathic thrombocytopenic purpura | SNOMED |
| 4133984 | Alloimmune thrombocytopenia | SNOMED |
| 4133983 | Secondary autoimmune thrombocytopenia | SNOMED |
| 4133981 | Benign gestational thrombocytopenia | SNOMED |
| 4125496 | Pure red cell aplasia, acquired | SNOMED |
| 4125494 | Pancytopenia with pancreatitis | SNOMED |
| 4123076 | Montreal platelet syndrome | SNOMED |
| 4123075 | May-Hegglin anomaly | SNOMED |
| 4123074 | Megakaryocytic thrombocytopenia | SNOMED |
| 4121265 | Mediterranean thrombocytopenia | SNOMED |
| 4121264 | Epstein syndrome | SNOMED |
| 4120620 | Amegakaryocytic thrombocytopenia | SNOMED |
| 4119134 | Thrombocytopenic purpura | SNOMED |
| 4103532 | Immune thrombocytopenia | SNOMED |
| 4102469 | Acute idiopathic thrombocytopenic purpura | SNOMED |
| 4101603 | Thrombocytopenia due to extracorporeal circulation of blood | SNOMED |
| 4101583 | Aplastic anemia due to infection | SNOMED |
| 4101582 | Aplastic anemia due to chronic disease | SNOMED |
| 4100998 | Aplastic anemia due to toxic cause | SNOMED |
| 4098148 | Thrombocytopenia due to drugs | SNOMED |
| 4098145 | Idiopathic aplastic anemia | SNOMED |
| 4098028 | Transient acquired pure red cell aplasia | SNOMED |
| 4098027 | Aplastic anemia due to radiation | SNOMED |
| 4082738 | Autoimmune pancytopenia | SNOMED |
| 4077348 | Pancytopenia-dysmelia | SNOMED |
| 4031699 | Humoral immunologic aplastic anemia | SNOMED |
| 4028065 | Autoimmune thrombocytopenia | SNOMED |
| 4027374 | Alloimmune platelet transfusion refractoriness | SNOMED |
| 4009307 | Heparin-induced thrombocytopenia with thrombosis | SNOMED |
| 4000065 | Drug-induced immune thrombocytopenia | SNOMED |
| 441264 | Primary thrombocytopenia | SNOMED |
| 440982 | Wiskott-Aldrich syndrome | SNOMED |
| 440372 | Acquired thrombocytopenia | SNOMED |
| 436956 | Evans syndrome | SNOMED |
| 433749 | Heparin-induced thrombocytopenia | SNOMED |
| 432881 | Pancytopenia | SNOMED |
| 318397 | Chronic idiopathic thrombocytopenic purpura | SNOMED |
| 140681 | Constitutional aplastic anemia | SNOMED |
| 138723 | Acquired red cell aplasia | SNOMED |
| 137829 | Aplastic anemia | SNOMED |

- - Thrombocytopenic purpura

| Concept ID | Concept name | Vocabulary |
| --- | --- | --- |
| 4119134 | Thrombocytopenic purpura | SNOMED |
| 4301602 | Thrombotic thrombocytopenic purpura | SNOMED |
| 4299560 | Thrombocytopenic purpura due to defective platelet production | SNOMED |
| 4292531 | Thrombocytopenic purpura due to platelet consumption | SNOMED |
| 4258261 | Drug induced thrombotic thrombocytopenic purpura | SNOMED |
| 4247776 | Posttransfusion purpura | SNOMED |
| 4230266 | Autoimmune thrombotic thrombocytopenic purpura | SNOMED |
| 4214947 | Thrombocytopenic purpura associated with metabolic disorder | SNOMED |
| 4204900 | Acquired thrombotic thrombocytopenic purpura | SNOMED |
| 4159966 | Upshaw-Schulman syndrome | SNOMED |
| 4140545 | Post infectious thrombocytopenic purpura | SNOMED |
| 4137430 | Idiopathic thrombocytopenic purpura | SNOMED |
| 4102469 | Acute idiopathic thrombocytopenic purpura | SNOMED |
| 318397 | Chronic idiopathic thrombocytopenic purpura | SNOMED |
| 313800 | Thrombotic microangiopathy | SNOMED |

- - Immune thrombocytopenia

| Concept ID | Concept name | Vocabulary |
| --- | --- | --- |
| 4103532 | Immune thrombocytopenia | SNOMED |
| 4137430 | Idiopathic thrombocytopenic purpura | SNOMED |
| 4133984 | Alloimmune thrombocytopenia | SNOMED |
| 4133983 | Secondary autoimmune thrombocytopenia | SNOMED |
| 4102469 | Acute idiopathic thrombocytopenic purpura | SNOMED |
| 4028065 | Autoimmune thrombocytopenia | SNOMED |
| 4027374 | Alloimmune platelet transfusion refractoriness | SNOMED |
| 4009307 | Heparin-induced thrombocytopenia with thrombosis | SNOMED |
| 4000065 | Drug-induced immune thrombocytopenia | SNOMED |
| 436956 | Evans syndrome | SNOMED |
| 433749 | Heparin-induced thrombocytopenia | SNOMED |
| 318397 | Chronic idiopathic thrombocytopenic purpura | SNOMED |

List of COVID-19 vaccinations:

| Concept ID | Name | Vocabulary |
| --- | --- | --- |
| 59267100003 | SARS-CoV-2 (COVID-19) vaccine, mRNA-BNT162b2 0.1 MG/ML Injectable Suspension | NDC |
| 59267100002 | SARS-CoV-2 (COVID-19) vaccine, mRNA-BNT162b2 0.1 MG/ML Injectable Suspension | NDC |
| 592671000 | bnt162b2 .23mg/1.8mL INTRAMUSCULAR INJECTION, SUSPENSION | NDC |
| 80777027310 | SARS-CoV-2 (COVID-19) vaccine, mRNA-1273 0.2 MG/ML Injectable Suspension | NDC |
| 2470234 | SARS-CoV-2 (COVID-19) vaccine, mRNA-1273 0.2 MG/ML Injectable Suspension | RxNorm |
| 2470233 | SARS-CoV-2 (COVID-19) vaccine, mRNA-1273 0.2 MG/ML | RxNorm |
| 2470232 | SARS-CoV-2 (COVID-19) vaccine, mRNA-1273 | RxNorm |
| 2468235 | SARS-CoV-2 (COVID-19) vaccine, mRNA-BNT162b2 0.1 MG/ML Injectable Suspension | RxNorm |
| 2468234 | SARS-CoV-2 (COVID-19) vaccine, mRNA spike protein Injectable Suspension | RxNorm |
| 2468233 | SARS-CoV-2 (COVID-19) vaccine, mRNA spike protein Injectable Product | RxNorm |
| 2468232 | SARS-CoV-2 (COVID-19) vaccine, mRNA-BNT162b2 0.1 MG/ML | RxNorm |
| 2468231 | SARS-CoV-2 (COVID-19) vaccine, mRNA spike protein | RxNorm |
| 2468230 | SARS-CoV-2 (COVID-19) vaccine, mRNA-BNT162b2 | RxNorm |
| 80777027399 | SARS-CoV-2 (COVID-19) vaccine, mRNA-1273 0.2 MG/ML Injectable Suspension | NDC |
| 807770273 | cx-024414 .2mg/mL INTRAMUSCULAR INJECTION, SUSPENSION | NDC |
| 39214411000001100 | Generic COVID-19 mRNA Vaccine BNT162b2 30micrograms/0.3ml dose concentrate for suspension for injection multidose vials (Pfizer-BioNTech) 1170 dose | dm+d |
| 39326611000001100 | Generic COVID-19 mRNA (nucleoside modified) Vaccine Moderna 0.1mg/0.5mL dose dispersion for injection multidose vials 100 dose | dm+d |
| 39326811000001100 | Generic COVID-19 mRNA (nucleoside modified) Vaccine Moderna 0.1mg/0.5mL dose dispersion for injection multidose vials | dm+d |
| 39214511000001100 | COVID-19 mRNA Vaccine BNT162b2 30micrograms/0.3ml dose concentrate for suspension for injection multidose vials (Pfizer-BioNTech) (Pfizer-BioNTech) 1170 dose 195 x 6 dose vials | dm+d |
| 39327011000001100 | COVID-19 mRNA (nucleoside modified) Vaccine Moderna 0.1mg/0.5mL dose dispersion for injection multidose vials (Moderna, Inc) 100 dose 10 x 10 dose vials | dm+d |
| 39326911000001100 | COVID-19 mRNA (nucleoside modified) Vaccine Moderna 0.1mg/0.5mL dose dispersion for injection multidose vials | dm+d |
| 39115611000001100 | COVID-19 mRNA Vaccine BNT162b2 30micrograms/0.3ml dose concentrate for suspension for injection multidose vials (Pfizer-BioNTech) | dm+d |
| 39115311000001100 | Generic COVID-19 mRNA Vaccine BNT162b2 30micrograms/0.3ml dose concentrate for suspension for injection multidose vials (Pfizer-BioNTech) 6 dose | dm+d |
| 39115711000001100 | COVID-19 mRNA Vaccine BNT162b2 30micrograms/0.3ml dose concentrate for suspension for injection multidose vials (Pfizer-BioNTech) (Pfizer-BioNTech) 6 dose | dm+d |
| 39116111000001100 | Generic COVID-19 mRNA Vaccine BNT162b2 30micrograms/0.3ml dose concentrate for suspension for injection multidose vials (Pfizer-BioNTech) | dm+d |
| 59676058005 | Janssen COVID-19 vaccine, DNA, spike protein, adenovirus type 26 (Ad26) vector, preservative free, 5x1010 viral particles/0.5mL dosage, for intramuscular use | NDC |
| 310122210 | AZD1222 Astrazeneca COVID-19 vaccine, DNA, spike protein, chimpanzee adenovirus Oxford 1 (ChAdOx1) vector, preservative free, 5x1010 viral particles/0.5mL dosage, for intramuscular use | NDC |
| 310122215 | azd1222 50000000000[VP]/.5mL INTRAMUSCULAR INJECTION, SUSPENSION | NDC |
| 59267100001 | SARS-CoV-2 (COVID-19) vaccine, mRNA-BNT162b2 0.1 MG/ML Injectable Suspension | NDC |
| 91303 | Janssen Covid-19 Vaccine | CPT4 |
| 91302 | AstraZeneca Covid-19 Vaccine | CPT4 |
| 91301 | Moderna Covid-19 Vaccine | CPT4 |
| 91300 | Pfizer-Biontech Covid-19 Vaccine | CPT4 |
| 0022A | AstraZeneca Covid-19 Vaccine Administration - Second Dose | CPT4 |
| 0021A | AstraZeneca Covid-19 Vaccine Administration - First Dose | CPT4 |
| 0031A | Janssen Covid-19 Vaccine Administration | CPT4 |
| 0012A | Moderna Covid-19 Vaccine Administration - Second Dose | CPT4 |
| 0011A | Moderna Covid-19 Vaccine Administration - First Dose | CPT4 |
| 0002A | Pfizer-Biontech Covid-19 Vaccine Administration - Second Dose | CPT4 |
| 0001A | Pfizer-Biontech Covid-19 Vaccine Administration - First Dose | CPT4 |
| 208 | SARS-COV-2 (COVID-19) vaccine, mRNA, spike protein, LNP, preservative free, 30 mcg/0.3mL dose | CVX |
| 207 | SARS-COV-2 (COVID-19) vaccine, mRNA, spike protein, LNP, preservative free, 100 mcg/0.5mL dose | CVX |
| 210 | SARS-COV-2 (COVID-19) vaccine, vector non-replicating, recombinant spike protein-ChAdOx1, preservative free, 0.5 mL | CVX |
| 212 | SARS-COV-2 (COVID-19) vaccine, vector non-replicating, recombinant spike protein-Ad26, preservative free, 0.5 mL | CVX |

**Treatments of interest**

| **Treatment of interest** | **Concept IDs** |
| --- | --- |
| Systemic corticosteroids (H02AB) | 21602729,21602731, 21602730, 21602733, 21602732, 21602735, 21602734, 21602744, 21602737, 21602736, 21602739, 21602738, 21602741, 21602740, 21602743, 21602742 |
| Vitamin K antagonists (B01AA) | 40798763, 1325124, 19024063, 19018364, 21600963, 19113013, 1310149, 19035344, 19033934, 40252605 |
| Heparins (B01AB) | 43009029, 19008276, 19026343, 1301025, 1301065, 21600973, 21600983, 19129274, 1436169, 19001014, 1308473 |
| Platelet aggregation inhibitors (B01AC) | 1327256, 1344992, 40241186, 35604848, 40798706, 1331270, 19042778, 1350310, 21600991, 21601002, 40163718, 46275677, 1302398, 19047423, 1354118, 19017067, 1322199, 1322184 |
| Thrombolytic/fibrinolytic enzymes (B01AD) | 19024191, 19098548, 19044890, 1307515, 19024544, 21601011, 21601012, 21601014, 1731597, 42801108, 1347450, 19136187 |
| Thrombolytic procedures | 2786475, 2786991, 2786476, 2786722, 2786723, 2787194, 2787193, 2787198, 2787199, 2786940, 2786941, 2786226, 2786225, 2787189, 2786447, 2787203, 2008245, 2786694, 2786695, 2787204, 2786968, 2786969, 2786195, 2786448, 2786196 |
| Dabigatran (B01AE07) | 40228152 |
| Direct thrombin inhibitors excl. dabigatran | 19084670, 40798953, 1322207, 19011712, 40799186, 19092139 |
| Direct factor Xa inhibitors (B01AF) | 43013024, 45892847, 1592988, 40241331 |
| Other anticoagulants (B01AX) | 1315865, 1366428, 21601029, 42898933 |
| Rituximab (L01XC02) | 1314273, 46275083, 46275082, 46275081, 46275080, 46275079, 46275078, 46275077, 46275076, 44195387, 44193088, 44120317, 44092633, 44088035, 44083996, 44072730, 44043697, 44036306, 43520205, 43520204, 43295512, 43284684, 43273901, 43214814, 43203967, 43203966, 43203965, 43203964, 43192931, 43192930, 43192929, 43181966, 43181965, 43160049, 43160048, 43160047, 43160046, 43160045, 43160044, 43148861, 43148860, 43148859, 43148858, 43137931, 43042670, 43041068, 43028136, 43028135, 43028133, 43028132, 43028131, 43028130, 43028129, 43028128, 43028127, 43028126, 43028125, 43028124, 43028123, 42958770, 42958769, 42958768, 42920725, 42920544, 42920543, 42919950, 42876643, 42876642, 42658425, 42482518, 41450305, 41450304, 41450303, 41450302, 41401180, 41400816, 41400110, 41399526, 41399427, 41399130, 41398728, 41398727, 41397332, 41397035, 41397034, 41396687, 41396144, 41394734, 41392650, 41391971, 41391284, 41391283, 41390646, 41373220, 41373219, 41371860, 41370629, 41370628, 41369440, 41364876, 41364875, 41364310, 41302142, 41295933, 41277369, 41246493, 41246492, 41237339, 41215489, 41090379, 41083973, 41058808, 41021177, 40956132, 40902903, 40834349, 40744310, 40744309, 40744308, 40744307, 40743660, 40743659, 40743658, 40715113, 40715112, 40715111, 40715110, 40715109, 40715108, 40715107, 40714100, 40714099, 40714098, 40714097, 40714096, 40714095, 40714094, 40714093, 40713601, 40713600, 37593999, 37593928, 37499798, 37499797, 37499796, 37499795, 37498582, 37498581, 37498580, 37498579, 37498578, 37498576, 36883074, 36813194, 36812426, 36812425, 36810430, 36810000, 36809986, 36809985, 36809981, 36808965, 36808958, 36808935, 36808892, 36787165, 36787164, 36787163, 36787162, 36787161, 36787160, 36787159, 36787158, 36787157, 36787156, 36787155, 36787079, 36780230, 36780229, 36780228, 36780227, 36780226, 36780225, 36780224, 36780223, 36779995, 36779994, 36779993, 36779188, 36779187, 36779186, 36779185, 36779184, 36779183, 36779182, 36779181, 36508812, 36507855, 36506757, 36506030, 36505789, 36505219, 36503610, 36503385, 36242524, 36227605, 35790889, 35790883, 35784602, 35782659, 35144548, 35135650, 35130468, 21177642, 21176809, 21167028, 21161314, 21161313, 21148180, 21147283, 21141572, 21138314, 21137006, 21131610, 21112147, 21107640, 21107639, 21099141, 21092455, 21082645, 21082644, 21072982, 21069754, 21068422, 21058945, 21049183, 21033535, 21033534, 21030372, 21029097, 21023767, 21023766, 19120213, 1593161, 1593160, 1593159, 1593158, 1593157, 1593156, 1593155, 1593154, 1593152, 1593151, 1593150, 1593149, 1356446, 1356445, 1356444, 1356443, 1355791, 1355790, 1355789, 1355788, 1355787, 1355785, 1314276, 784803, 784548 |
| Fibrinogen (B02BB) | 21601048, 190547 |
| Immunoglobulins (J06B) | 21601254 |
| Plasma exchange/platelet transfusion (procedures, B05AX03) | 21601151, 4028665, 4121918, 4118896, 4022171, 4024248 |

Details of Concept IDs can be found on <https://athena.ohdsi.org/>.

**SUPPLEMENTARY MATERIAL C**

**Study settings treatment pathways**

| **General settings** | | |
| --- | --- | --- |
| studyName | Unique name identifying the set of study parameters below | Main analysis |
| targetCohortId | Select one study population | VTE, ATE or TTS |
| eventCohortIds | Select all treatments of interest | All treatments of interest |
| **Analysis settings** | | |
| includeTreatmentsPriorToIndex | Number of days prior to the index date of the target cohort that event cohorts are allowed to start | 0 |
| minEraDuration | Minimum time an event era should last to be included in analysis | 0 |
| eraCollapseSize | Window of time between which two eras of the same event cohort are collapsed into one era | 7 |
| combinationWindow | Window of time two event cohorts need to overlap to be considered a combination treatment | 1 |
| minPostCombinationDuration | Minimum time an event era before or after a generated combination treatment should last to be included in analysis | 1 |
| filterTreatments | Select first occurrences of / changes between / all event cohorts | All |
| **Output settings** | | |
| maxPathLength | Maximum number of steps included in treatment pathway | 5 |
| minCellCount | Minimum number of persons with a specific treatment pathway for the pathway to be included in analysis | 5 |
| minCellMethod | Select to completely remove / sequentially adjust (by removing last step as often as necessary) treatment pathways below minCellCount | Adjust |
| groupCombinations | Select to group all non-fixed combinations in one category ‘other’ in the sunburst plot | 5 |
| addNoPaths | Select to include untreated persons without treatment pathway in the sunburst plot | FALSE |

**SUPPLEMENTARY MATERIAL D**

**Results for post-vaccine period population cohort**

Baseline characteristics of post-vaccine period TTS, VTE and ATE patients.

|  | **Characteristic** | **Hospital CDM (USA)** | **IMASIS**  **(ES)** | **CPRD (UK)** | **SIDIAP (ES)** | **IPCI (NL)** | **LPD France (FR)** | **DA Germany (DE)** | **Open Claims (USA)** |
| --- | --- | --- | --- | --- | --- | --- | --- | --- | --- |
| TTS | Number of patients | 7,852 | 140 | 218 | 1,440 | 8 | 8 | 126 | 36,005 |
|  | Treated, % | 40.4 | 77.9 | 7.3 | 34.0 | 37.5 | 62.5 | 20.6 | 8.7 |
|  | Gender: Male, % | 58.1 | 65.7 | 69.7 | 68.7 | 87.5 | 100 | 65.9 | 56.4 |
|  | Age at index (years), Mean (SD) | 66.9  (13.0) | 73.2  (14.3) | 72.4  (15.0) | 73.8  (13.4) | 68.4  (16.3) | 72.4  (11.5) | 72.0  (13.1) | 67.4  (14.3) |
|  | Charlson comorbidity index, Mean (SD) | 6.5  (3.7) | 3.4  (3.2) | 1.8  (2.2) | 4.8  (3.4) | 2.0  (2.0) | 1.8  (1.5) | 4.6  (3.2) | 6.5  (3.9) |
| VTE | Number of patients | 83,613 | 382 | 16,199 | 6,496 | 3,980 | 8,719 | 15,232 | 1,581,502 |
|  | Treated, % | 25.3 | 61.3 | 11.0 | 51.1 | 24.6 | 43.3 | 28.6 | 23.8 |
|  | Gender: Male, % | 48.4 | 55 | 50.9 | 50.7 | 45.3 | 49.5 | 46.5 | 48.3 |
|  | Age at index (years), Mean (SD) | 63.5  (14.5) | 69.2  (16.0) | 63.6  (16.9) | 69.2  (15.6) | 62.5  (15.7) | 70.6  (14.2) | 68.0  (15.4) | 64.4  (15.4) |
|  | Charlson comorbidity index, Mean (SD) | 3.9  (3.6) | 2.2  (2.8) | 1.8  (2.2) | 3.0  (3.1) | 1.5  (1.6) | 1.0  (1.4) | 2.8  (2.9) | 4.3  (3.5) |
| ATE | Number of patients | 156,863 | 732 | 17,832 | 15,933 | 19,207 | 38,320 | 28,415 | 4,745,099 |
|  | Treated, % | 26.7 | 58.9 | 3.8 | 33.4 | 7.5 | 38.4 | 10.6 | 7.3 |
|  | Gender: Male, % | 55.0 | 58.7 | 67.1 | 59.5 | 65.6 | 73.0 | 62.9 | 51.3 |
|  | Age at index (years), Mean (SD) | 67.2  (12.2) | 72.9  (14.2) | 67.4  (13.4) | 72.7  (13.9) | 69.7  (11.9) | 69.-  (12.4) | 69.7  (13.2) | 69.5  (13.0) |
|  | Charlson comorbidity index, Mean (SD) | 4.9  (3.4) | 2.7  (3.0) | 2.6  (2.0) | 3.7  (2.9) | 2.5  (1.7) | 1.8  (1.3) | 3.8  (2.9) | 5.8  (3.6) |


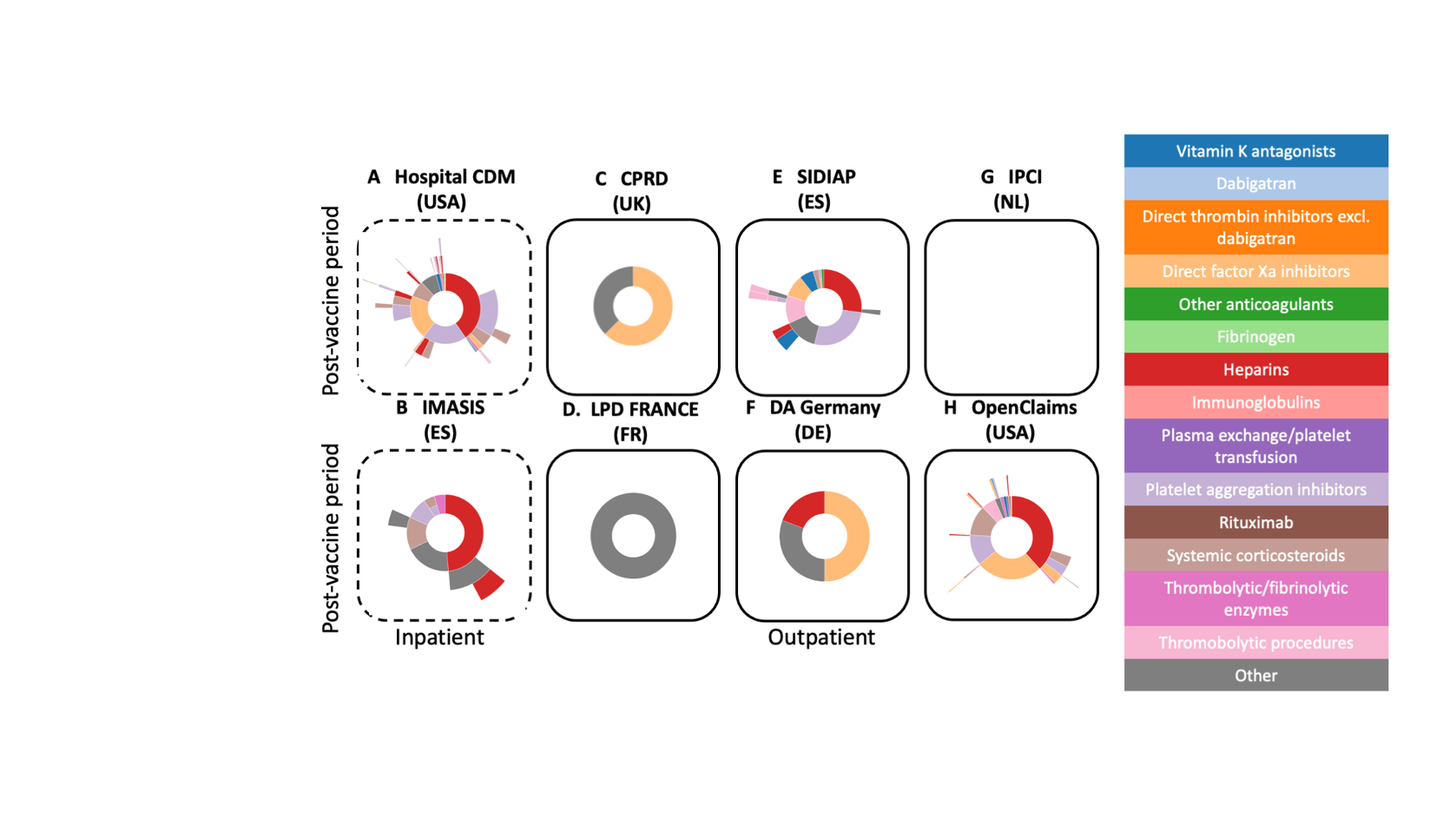


Figure S1: Sunburst plots visualizing treatment pathways for TTS patients in post-vaccine period cohorts. Inpatient databases are depicted with a dashed line frame, whilst outpatient ones have a solid frame.


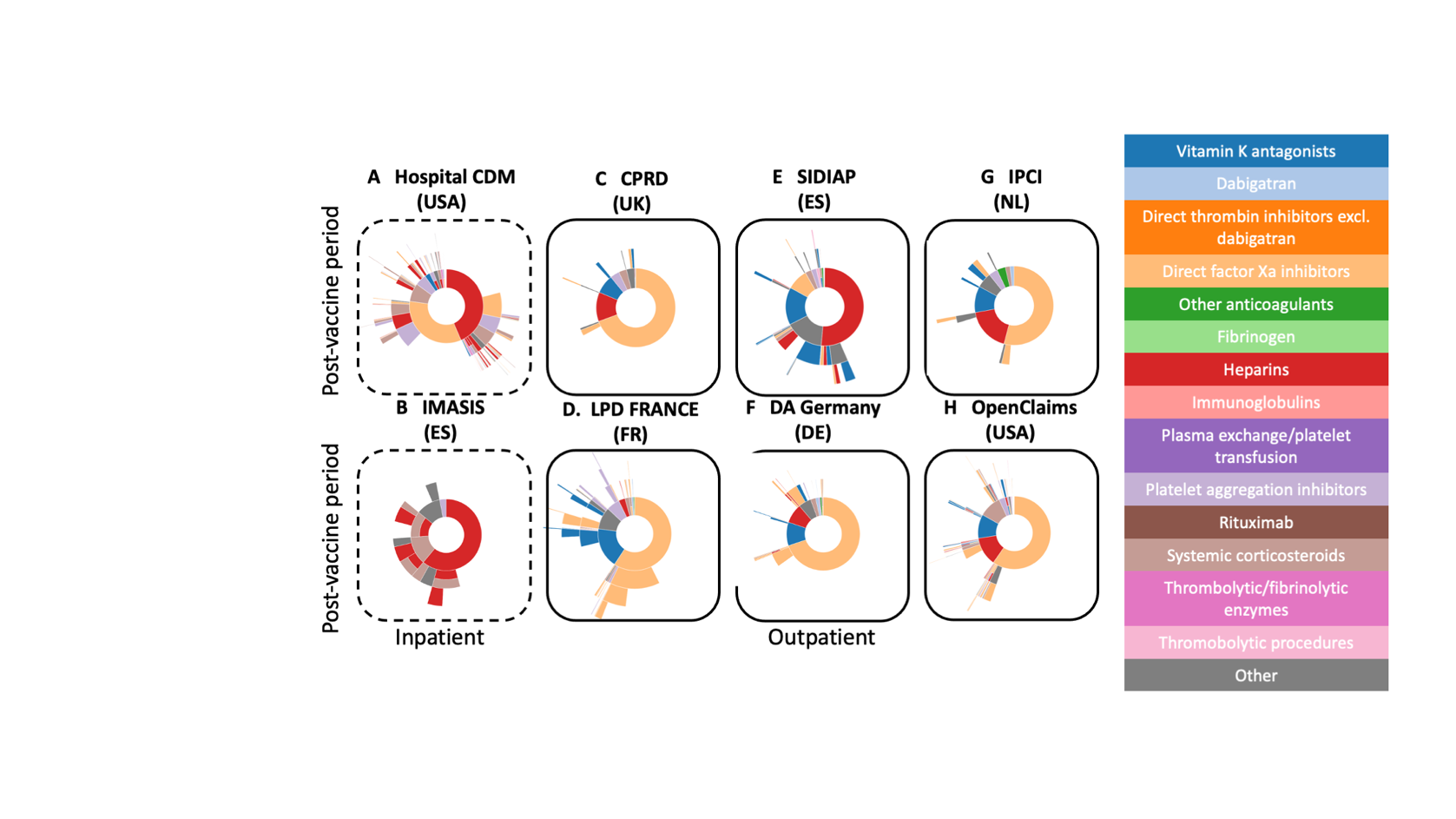


Figure S2: Sunburst plots visualizing treatment pathways for VTE patients in post-vaccine period cohorts. Inpatient databases are depicted with a dashed line frame, whilst outpatient ones have a solid frame.


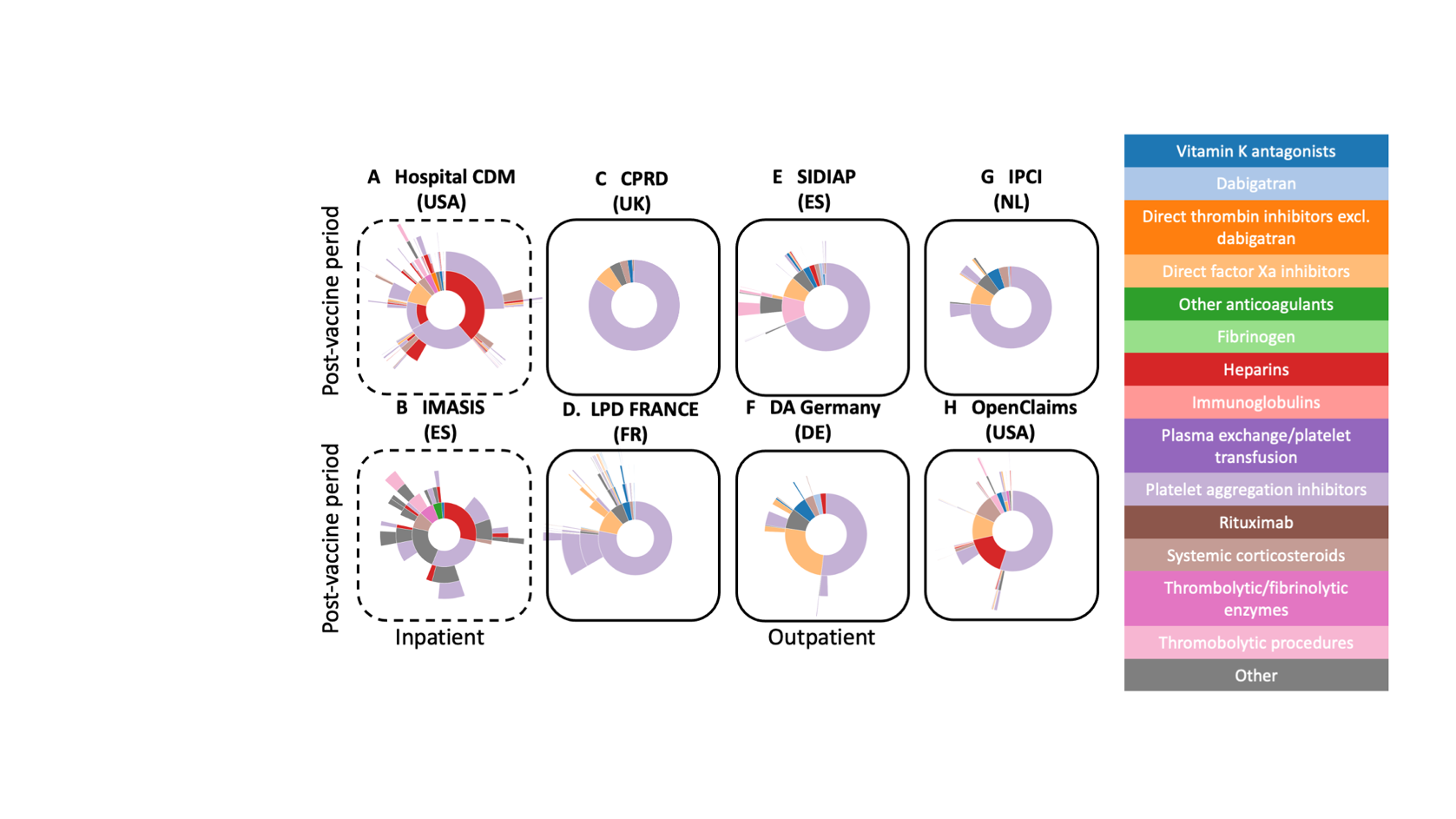


Figure S3: Sunburst plots visualizing treatment pathways for ATE patients in post-vaccine period cohorts. Inpatient databases are depicted with a dashed line frame, whilst outpatient ones have a solid frame.
